# Caloric Restriction Mimetic Hydroxycitrate Mitigates Acute Nephrotoxicity via Autophagy Activation and Oxidative Stress Reduction

**DOI:** 10.3390/biom16040538

**Published:** 2026-04-04

**Authors:** Xinyu Liao, Nadezda V. Andrianova, Ljubava D. Zorova, Anna A. Brezgunova, Kseniia S. Cherkesova, Marina I. Buyan, Dmitry S. Semenovich, Alexandra A. Dalina, Irina B. Pevzner, Juan Jin, Yunguang Wang, Egor Y. Plotnikov

**Affiliations:** 1 A.N. Belozersky Institute of Physico-Chemical Biology, Lomonosov Moscow State University, 119992 Moscow, Russia; starrain524@gmail.com (X.L.); andrianova@belozersky.msu.ru (N.V.A.); ljuzor@belozersky.msu.ru (L.D.Z.); cherkesova.ks@mail.ru (K.S.C.); marinanenart@gmail.com (M.I.B.); 7emenovich@gmail.com (D.S.S.); pevzner_ib@belozersky.msu.ru (I.B.P.); 2 Faculty of Bioengineering and Bioinformatics, Lomonosov Moscow State University, 119234 Moscow, Russia; 3 Faculty of Biology, Lomonosov Moscow State University, 119234 Moscow, Russia; 4 Engelhardt Institute of Molecular Biology, Russian Academy of Sciences, 119991 Moscow, Russia; alexandra.dalina@gmail.com; 5 Department of Nephrology, Zhejiang Provincial Hospital of Chinese Medicine, Hangzhou 310006, China; 20233004@zcmu.edu.cn (J.J.); wordlist@aliyun.com (Y.W.)

**Keywords:** caloric restriction, caloric restriction mimetics, acute kidney injury, inflammation, autophagy, mitochondria, oxidative stress, apoptosis

## Abstract

Drug-induced nephrotoxicity is a leading cause of acute kidney injury (AKI) and subsequent chronic kidney disease. Nephrotoxicity often develops as a consequence of treatment with commonly prescribed aminoglycoside antibiotics, and remains a significant clinical challenge. One approach to treating AKI and its associated complications is caloric restriction or its pharmacological mimetics. This study aimed to evaluate the effects of caloric restriction mimetic hydroxycitrate (HC) in gentamicin-induced nephrotoxicity, with particular focus on the influence of treatment duration and the underlying molecular mechanisms. In vitro renal tubular epithelial cells models were used to assess HC’s effects on viability, proliferation, and autophagy activation. For in vivo validation, rats with gentamicin-induced AKI received HC treatment via two distinct regimens (3-week and 7-week administration). Experiments on renal tubule cells showed that HC significantly increased cell viability and proliferation and led to the activation of autophagy. In the rat model, only the 7-week administration of HC demonstrated significantly attenuated renal dysfunction in gentamicin-induced AKI. Moreover, it reduced macrophage infiltration, increased renal cell tolerance to apoptosis, activated autophagy, and reduced oxidative stress. Thus, our results indicate that 7-week HC administration could be used as a prophylactic strategy against antibiotic nephrotoxicity, exerting its effects by promoting autophagy, resisting apoptosis, and attenuating oxidative damage.

## 1. Introduction

Acute kidney injury (AKI) comprises a group of syndromes and has become a worldwide health problem that endangers patients in hospitals and intensive care units (ICUs), and which can also threaten healthy individuals [1,2]. AKI represents a significant clinical challenge, characterized by an abrupt decline in kidney function [3]. The reported incidence of AKI is approximately 2.1 per 1000 of the population [4,5]; in-hospital mortality is 24% and increases with the severity of the disease [6,7,8]. In elderly patients, mortality can reach 57% [9]; in some situations, AKI progresses to chronic kidney disease [10], which increases the risk of cardiovascular diseases [11] and sudden cardiac death [12].

Among the various causes of AKI, nephrotoxic drugs, such as aminoglycoside antibiotics, play a considerable role [13]. Gentamicin, a widely used aminoglycoside, is particularly known for its nephrotoxic potential, contributing significantly to the incidence of drug-induced AKI [14]. Despite its therapeutic efficacy in treating severe bacterial infections, the adverse effects of gentamicin on the kidneys have prompted the search for protective strategies and agents [15]. Nephrotoxicity of gentamicin is caused by its selective accumulation in renal tubules, initiated by megalin-mediated endocytosis, followed by a cascade of damaging events, leading to the death of tubular epithelial cells [16]. Molecular mechanisms of gentamicin-induced nephrotoxicity are based on the dysregulation of autophagy, lysosomal impairment, direct damage to mitochondrial electron transport chain complexes, resulting in reactive oxygen species overproduction, and the activation of NF-κB-driven inflammation [17].

Numerous studies have extensively documented that caloric restriction has a protective effect against AKI as well as in a variety of other pathological conditions [18,19,20]. However, in both prevention and therapy, caloric restriction is usually difficult for patients to follow, so drugs that activate the same signaling pathways are being actively studied. In this context, the effects and molecular mechanisms of caloric restriction and its pharmacologic mimetics in various pathologies are extensively investigated [21]. Hydroxycitrate (HC), a competitive inhibitor of ATP–citrate lyase, has attracted attention due to its potential therapeutic properties [22]. Known primarily for its role in weight loss and metabolism regulation, HC also exhibits reported antioxidant [23] and anti-inflammatory [24] effects. In addition, recent studies have shown that autophagy is an effective therapeutic target [25,26,27,28,29], and HC has been used as an autophagy activator in various diseases [30,31]. These properties suggest a potential for nephroprotection, particularly against drugs with nephrotoxic side effects like gentamicin that induce oxidative stress and inflammation in kidney tissue [32,33,34,35,36].

Despite preliminary evidence for the beneficial effects of HC, the specific mechanisms by which HC might attenuate nephrotoxicity, particularly in the context of gentamicin-induced AKI, and the optimal duration of HC treatment as a determining factor remain understudied. The aim of this study was to investigate, both in vitro and in vivo, the protective effects of HC during gentamicin-induced AKI, focusing on its ability to attenuate oxidative stress, inflammation, and cell death in renal tissue. In particular, we compared the influence of the duration of HC treatment, a factor that has been underestimated in previous research.

## 2. Materials and Methods

### 2.1. Cell Cultures

NRK-52E cells from a rat kidney epithelial cell line (CL-0174, SYNTHBIO, Ltd., Hefei, China) were cultured in a culture medium consisting of DMEM/F-12 (PanEco, Moscow, Russia) supplemented with 10% fetal bovine serum (FBS) (BioSera, Cholet, France) and 1% L-glutamine (PanEco, Moscow, Russia). The cells were maintained at 37 °C in a humid atmosphere containing 5% CO_2_. Cells were passaged every 3–4 days at approximately 80–90% confluence. Prior to treatment, cells were seeded in appropriate culture plates and allowed to adhere overnight. For transfection, MDCK cell line (CCL-34, ATCC, Manassas, VA, USA) was used. MDCK was cultured in culture medium DMEM/F-12 (PanEco, Moscow, Russia) with 5% FBS (BioSera, Cholet, France) and 1% L-glutamine (PanEco, Moscow, Russia).

### 2.2. MTT Test

Cell viability was assessed using the MTT test [37] after 48 h of treatment with HC (Sigma-Aldrich, St. Louis, MO, USA) at concentrations of 0.0625–16 mM for NRK-52E cells and 0.001–20 mM for MDCK cells. For autophagy inhibitory analysis, NRK-52E cells were additionally incubated with 0.5 mM HC for 24 h in the presence of 3-methyladenine (3-MA, 2.5 mM, Sigma-Aldrich, St. Louis, MO, USA) or with chloroquine (CQ, 40 μM, Sigma-Aldrich, St. Louis, MO, USA). MTT solution (5 mg/mL in DMEM/F-12 without sodium bicarbonate; PanEco, Moscow, Russia) was added for 1 h at 37 °C, followed by washing and solubilization with dimethyl sulfoxide (DMSO). The absorbance was measured using a Zenyth 3100 plate multimode detector (Anthos Labtec, Salzburg, Austria) at 595 nm. Cell viability was calculated as the percentage of absorbance in treated wells relative to the control, which was set as 100% viability.

### 2.3. Real-Time Cell Proliferation Monitoring

Cell proliferation was estimated using the iCELLigence real-time cell analysis system (ACEA Biosciences Inc., San Diego, CA, USA). NRK-52E cells were grown on the plates for 24 h, then incubated with 1 mM HC in culture medium.

### 2.4. Autophagy Monitoring

To evaluate autophagy activation, NRK-52E cells were incubated with 1 mM HC for 24 h in a glass-bottom dish (MatTek, Ashland, MA, USA), washed with Dulbecco’s phosphate-buffered saline (DPBS) and loaded with 2 μM Cyto-ID (Enzo Life Sciences, Farmingdale, NY, USA). Cyto-ID fluorescence was evaluated using 488 nm excitation wavelength with emission collected at 500–530 nm using an LSM 900 confocal microscope (Zeiss, Oberkochen, Germany) provided by the Moscow State University Development Program.

To evaluate autophagy activation, MDCK cells stably expressing mCherry-green fluorescent protein (GFP)-light chain 3 (LC3) were obtained by lentiviral transduction. In brief, HEK293T cells were transfected with the plasmid pLX301-mCherry-GFP-LC3B (kindly provided by Dr. Ana Maria Cuervo), the lentiviral packaging plasmids encoding Pol, Rev, Gag, and the envelope plasmid encoding VSV-G. MDCK cells were infected with the obtained lentiviral particles and selected using puromycin (10 μg/mL for 24 h) (BioinnLabs, Moscow, Russia). After selection, MDCK cells were passed and then incubated with 1 mM HC for 48 h in glass-bottom dishes. GFP fluorescence was evaluated using 488 nm excitation wavelength with emission collected at 495–560 nm using an LSM 900 confocal microscope equipped with an Airyscan 2 detector for super-resolution (Zeiss, Germany), mCherry fluorescence was evaluated using 561 nm excitation wavelength with emission collected at 570–700 nm using an LSM 900 confocal microscope (Zeiss, Oberkochen, Germany). For measurement of total GFP and mCherry fluorescence, 15 fields of view for each experimental group were obtained on C-Apochromat 10×/0.45 objective, image analysis was performed using Fiji/ImageJ software (version 2.9.0) [38]. For analysis of GFP and mCherry colocalization, images of transfected MDCK cells were performed on Plan-Apochromat 63×/1.4 objective with the super-resolution module. GFP and mCherry fluorescence profiles were obtained using Zen software (version 3.4.91.00000, Zeiss, Germany).

For quantitative image analysis, cell segmentation was first performed on control and HC-treated (for 6 h) MDCK cells transfected with the mCherry-GFP-LC3 reporter using the deep-learning-based algorithm Cellpose 2.0 (cyto2 model) in Python (version 3.9) [39], applied to raw fluorescence images acquired in CZI format. Images were converted to 8-bit and intensity-normalized prior to segmentation. The Cellpose model was run with a fixed cell diameter parameter (empirically selected and validated by visual inspection), yielding labeled masks in which each individual cell was assigned a unique integer label.

Cell area was calculated as the number of pixels within each mask multiplied by the calibrated pixel area (0.001849 µm^2^ per pixel). The intracellular compartment mask (detecting autolysosomes) was generated using adaptive thresholding followed by binarization. Autolysosome area per cell was calculated as the intersection between the compartment mask and the corresponding cell mask and converted to µm^2^ using the same calibration factor.

### 2.5. Animals

Experiments were performed on 48 male Wistar rats (10–12 weeks old, 250–300 g). The animal protocols were approved by the Animal Ethics Committee of the A.N. Belozersky Institute of Physico-Chemical Biology Lomonosov Moscow State University (Protocol 006-1/2/2024 from 1 February 2024). All procedures were performed in accordance with the “Animal Research: Reporting of In Vivo Experiments” (ARRIVE) guidelines. Animals had unlimited access to food and water and were maintained in cages in a temperature-controlled environment (20 ± 1 °C) under a 12 h/12 h light/dark regime. The food intake of all experimental animals was measured every 2 days. Body weight was monitored weekly (Supplementary Materials, Figures S1 and S2).

### 2.6. HC Administration

**Figure 1:**
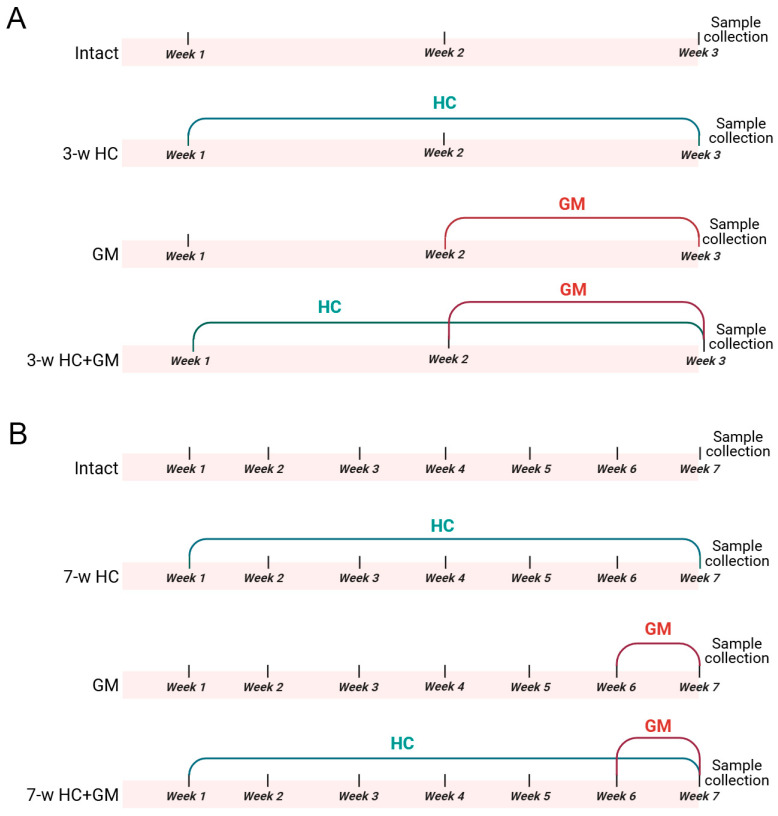
Experimental design: (**A**) 3-week-regimen HC administration, including intact, HC-treated (“3-w HC”), gentamicin-treated (“GM”) and HC + gentamicin-treated (“3-w HC + GM”) groups; (**B**) 7-week-regimen HC administration, including intact, HC-treated (“7-w HC”), gentamicin-treated (“GM”) and HC + gentamicin-treated (“7-w HC + GM”) groups. GM—gentamicin, HC—hydroxycitrate.

Two HC administration regimens were used: 3-week HC administration at a dose of 300 mg/kg/day (Figure 1A); 7-week HC administration at a dose of 600 mg/kg/day (Figure 1B). For the short-term regimen, rats were randomly divided into 4 groups: (i) intact (*n* = 4), (ii) HC-treated (“3-w HC”, *n* = 4), (iii) gentamicin-treated (“GM”, *n* = 8) and (iv) HC + gentamicin treated (“3-w HC + GM”, *n* = 8). The same was done for the long-term regimen, where the rats were randomly divided into 4 groups: (i) intact (*n* = 4), (ii) HC-treated (“7-w HC”, *n* = 4), (iii) gentamicin-treated (“GM”, *n* = 8) and (iv) HC + gentamicin treated (“7-w HC + GM”, *n* = 8). The rats in the HC-treated groups received HC in the form of Garcinia cambogia extract (NATROL Company, Chatsworth, CA, USA), which consists of 60% HC. HC was diluted in drinking water to 100 mg/mL and administered once daily by oral gavage. In the “HC + GM” groups, HC was administered 3 h before gentamicin injection.

### 2.7. Induction of AKI and Sample Collection

In the 3-week regimen, after 2 weeks of HC pretreatment, AKI was induced in “GM” and “HC + GM” groups by administering gentamicin (“Mosagrogen”, Moscow, Russia, 4%) via intraperitoneal injection (i.p.) at a dose of 160 mg/kg/day for 6 consecutive days. This dose and the duration were chosen to ensure the development of kidney injury [40,41]. In the 7-week regimen, after 6 weeks of HC pretreatment, AKI was induced in the “GM”- and “HC + GM”-treated groups by administering gentamicin i.p. at a dose of 160 mg/kg/day for 6 consecutive days.

Blood and urine samples were collected 24 h following 6 days of gentamicin administration. Kidney function was assessed by measuring serum creatinine (SCr) and blood urea nitrogen (BUN) in serum using the AU480 Chemistry System (Beckman Coulter, Brea, CA, USA) according to the manufacturer’s instructions. Kidneys were harvested for Western blotting and biochemical analysis to evaluate the severity of kidney injury and the protective effects of HC.

### 2.8. Western Blotting

Urine samples were centrifuged at 10,000× *g* for 5 min, then mixed with an equal volume of 2× sample buffer containing 10% 2-mercaptoethanol, followed by boiling for 5 min. Kidney tissues were homogenized in 5 mL phosphate-buffered saline (PBS) containing 1 mM protease inhibitor phenylmethylsulfonyl fluoride (PMSF), and subsequently centrifuged at 1000× *g* for 3 min. Protein concentration was determined using the bicinchoninic acid assay kit (Sigma Aldrich, St. Louis, MO, USA). Prior to gel electrophoresis, samples were centrifuged again at 10,000× *g* for 5 min. For urine samples, 20 μL of each sample was loaded per lane onto 15% Tris-glycine polyacrylamide gels, while for kidney samples, 10 μg of protein was loaded per lane. Following electrophoretic separation, proteins were transferred onto PVDF membranes (Amersham Pharmacia Biotech, Little Chalfont, Buckinghamshire, UK). Membranes were blocked with 5% non-fat milk in PBS containing 0.05% Tween-20, and then incubated with anti-neutrophil gelatinase-associated lipocalin (NGAL) 1:1000 rabbit (#AB63929, Abcam, Cambridge, UK), anti-kidney injury molecule-1 (KIM-1) (1:1000 mouse (#MAA785Ra21, Cloud Clone Corp., Katy, TX, USA), anti-proliferating cell nuclear antigen (PCNA) 1:1000 rabbit (#13110, Cell Signaling, Danvers, MA, USA), anti-B-cell lymphoma extra-large (Bcl-X_L_) 1:1000 rabbit (#2764, Cell Signaling, Danvers, MA, USA), anti-cluster of differentiation 68 (CD68) 1:1000 rabbit (#DF7518, Cell Signaling, Danvers, MA, USA), anti-beta-actin (β-actin) 1:2000 mouse (#A2228, Sigma-Aldrich, Danvers, MA, USA), anti-LC3-II/LC3-I 1:1000 rabbit (#12471, Cell Signaling, Danvers, MA, USA), anti-beclin-1 1:1000 rabbit (#3495, Cell Signaling, Danvers, MA, USA), anti-proliferator-activated receptor gamma coactivator 1-alpha (PGC-1α) rabbit 1:1000 (#PA5-38022, Invitrogen, Thermo Fisher Scientific, Carlsbad, CA, USA) primary antibodies. Subsequently, membranes were incubated with horseradish peroxidase-conjugated secondary anti-rabbit or anti-mouse antibodies (IMTEK, Moscow, Russia), developed using the Advansta Western Bright ECL kit (Advansta, San Jose, CA, USA). Protein bands were visualized using the V3 Western Blot Imager (BioRad, Hercules, CA, USA). Western blot signals were normalized to β-actin (kidney lysates) and to equal loading (urine). Original figures can be found in Supplementary Materials. 

### 2.9. Determination of Antioxidant Status Parameters

Kidney tissue was homogenized in 1× PBS pH 7.4 at a ratio of 1:10 (*w*/*v*) and centrifuged at 3000× *g* for 5 min at 4 °C. The supernatant was used to assess antioxidant status parameters and measure total protein using the Lowry method [42].

Total antioxidant activity in kidney homogenates was assessed by inhibition of the formation of thiobarbiturate-reactive substances (TBARS) upon induction of lipid peroxidation by 50 µM FeSO_4_ and 0.5 mM ascorbate. The samples were incubated at 37 °C for 1 h. The concentration of formed TBARS was determined spectrophotometrically at 532 nm [43] using a PE-5400UV spectrophotometer (“Ekrokhim” LLC, Saint Petersburg, Russia). The TBARS content was estimated from an extinction coefficient of 156 mM^−1^·cm^−1^ and expressed as μmol·h^−1^·mg^−1^·protein.

The total tissue thiol content in kidney homogenate was determined using a spectrophotometric method based on the reaction with Ellman’s reagent [43,44]. The absorbance was measured at 412 nm using a PE-5400UV spectrophotometer (“Ekrokhim” LLC, Saint Petersburg, Russia). The concentration of thiols was calculated using a molar extinction coefficient of 13.6 mM^−1^·cm^−1^, and the results were expressed as nmol/mg of protein.

### 2.10. Statistical Analysis

Data were presented as the mean ± standard error of the mean (SEM). The data were tested for normality using the Shapiro–Wilk test. For comparison of two groups, the *t*-test was used in case of parametric variables and the Mann–Whitney U-test in case of non-parametric variables. For comparison between several groups, one-way ANOVA with Tukey’s post hoc test or the Kruskal–Wallis test with Dunn’s test in case of non-normally distributed data were used. Outliers were removed from the analysis using ROUT method (Q = 1%). Data was analyzed using Microsoft Excel software (version KB4011684, Redmond, DC, USA) and GraphPad Prism (version 8, GraphPad Software Inc., San Diego, CA, USA).

## 3. Results

### 3.1. HC Enhanced Kidney Cell Viability and Proliferation

**Figure 2:**
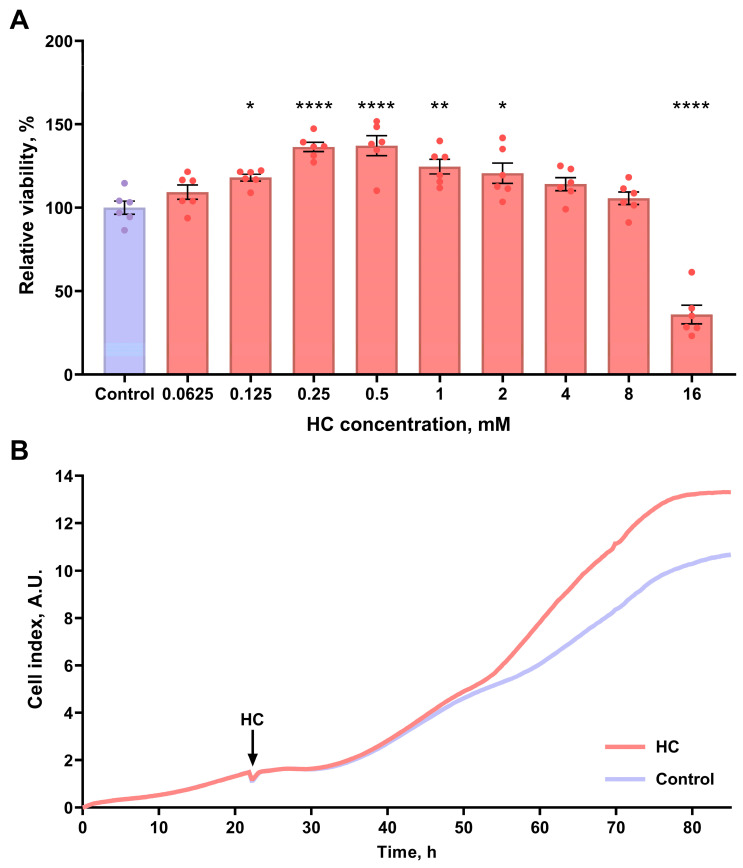
Effects of HC on the viability and proliferation of kidney cells in vitro. (**A**) Relative viability of NRK-52E assessed by MTT-test after incubation with different concentrations of HC, data are presented as percentage relative to the untreated control group (set as 100%); (**B**) the averaged growth curves of NRK-52E in standard conditions and exposed to 1 mM HC. Cell index values are presented in arbitrary units (A.U.). * *p* < 0.05, ** *p* < 0.01, **** *p* < 0.0001 compared to the control group (one-way ANOVA with Tukey’s post hoc test).

We assessed the in vitro effects of HC on kidney cell viability and proliferation. We demonstrated that incubation with HC at concentrations ranging from 0.125 mM to 2 mM significantly increased the MTT-test-estimated viability of NRK-52E kidney cells (Figure 2A). Furthermore, HC at concentrations of 0.5–10 mM also enhanced the viability of MDCK kidney cells (Supplementary Materials, Figure S3). This indicated increased proliferation of the cells, which was confirmed by real-time monitoring of cell index. Indeed, we found that 1 mM HC increased the proliferation rate of NRK-52E cells compared to the control group (Figure 2B).

### 3.2. HC Activated Autophagy in Kidney Cells

**Figure 3:**
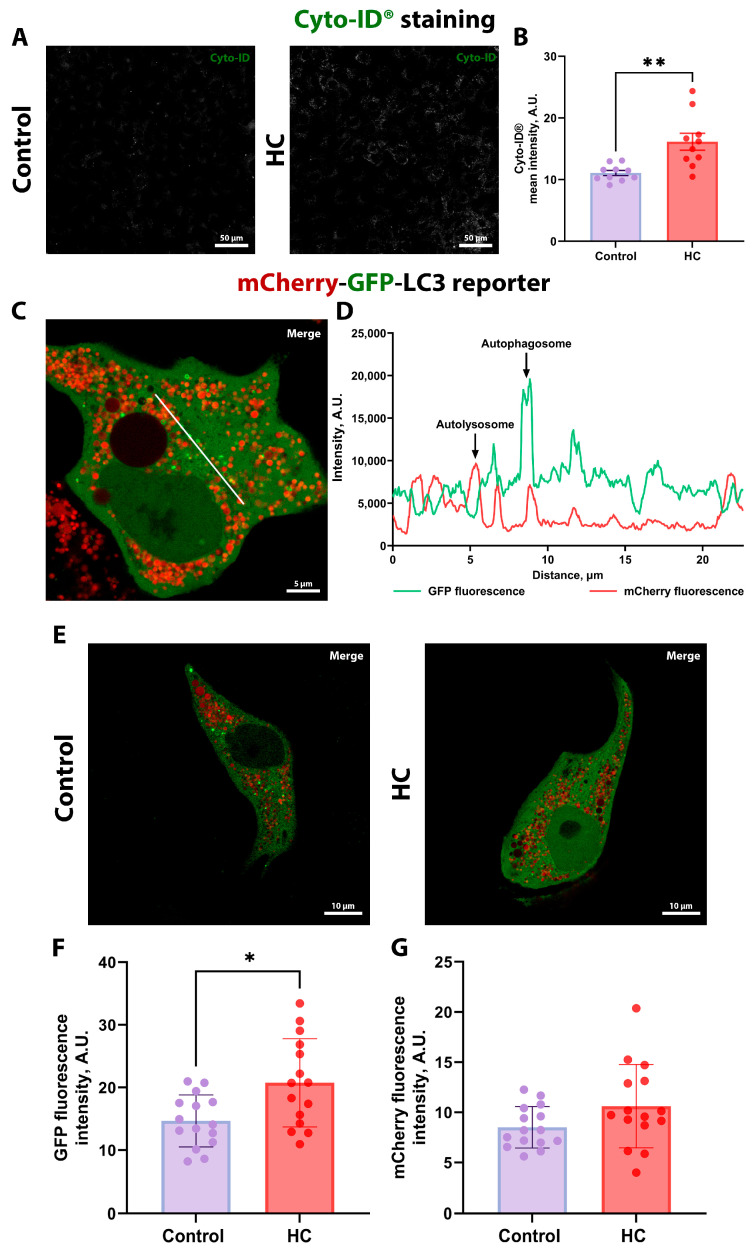
The assessment of autophagic activity of kidney cells in vitro. (**A**) Representative confocal images showing Cyto-ID fluorescence in control and HC-treated NRK-52E cells. Scale bar, 50 μm. (**B**) Quantitative image analysis of Cyto-ID fluorescence intensity in control and HC-treated NRK-52E cells. (**C**) Representative super-resolution Airyscan images of transfected MDCK with the mCherry-GFP-LC3 reporter, showing GFP and mCherry fluorescence in control cell. The white line indicates where GFP and mCherry signal profiles were measured and matched. Scale bar, 5 μm. (**D**) Colocalization analysis of GFP and mCherry signals in MDCK cells expressing the mCherry-GFP-LC3 reporter. (**E**) Representative confocal images showing mCherry and GFP fluorescence in control and HC-treated MDCK cells. Scale bar, 10 μm. (**F**) Quantitative image analysis of GFP fluorescence intensity in control and HC-treated MDCK cells. (**G**) Quantitative image analysis of mCherry fluorescence intensity in control and HC-treated MDCK cells. * *p* < 0.05, ** *p* < 0.01 ((**B**): unpaired *t*-test; (**F**,**G**): Mann–Whitney test).

The in vitro effect of HC on autophagy was analyzed in kidney cells using the Cyto-ID fluorescent probe, which allows selective detection of autophagosomes and autophagolysosomes [45]. HC treatment significantly increased Cyto-ID fluorescence intensity in NRK-52E cells (Figure 3A), as shown by quantitative analysis of fluorescence intensity (Figure 3B).

These results suggest that HC has the potential to promote autophagy in cells. MDCK kidney cells with stable expression of mCherry-GFP-LC3 reporter [46] were also used to assess autophagosome accumulation in cells after incubation with HC. The mCherry-GFP-LC3 fusion protein exists in three forms in the cell: in the cytoplasm after protein synthesis (characterized by both GFP and mCherry fluorescence), in early autophagosomes (both GFP and mCherry fluorescence), and in acidified autolysosomes (only mCherry fluorescence) (Figure 3C). To explore colocalization, we performed analysis of GFP and mCherry fluorescence profiles in the transfected MDCK cells, which demonstrated that, while some organelles exhibited both signals (representing pH-neutral autophagosomes that have not yet fused with lysosomes), the majority were red-only, confirming their identity as acidified autolysosomes (Figure 3C,D).

For analysis of HC effects, we obtained images of MDCK cells transfected with the mCherry-GFP-LC3 reporter at both high (Figure 3E; Supplementary Materials, Figure S4B,E) and low (Supplementary Materials, Figure S4A) magnifications to assess differences in LC3 expression and the number of autophagosomes/autolysosomes. After incubation with HC, we observed an increased GFP fluorescence (Figure 3F), indicating elevated LC3 expression and autophagosome formation. However, mCherry fluorescence did not significantly increase after incubation with HC (Figure 3G). To evaluate autolysosome content more precisely, we applied cell masks based on the cytoplasmic GFP channel to define whole-cell areas (Supplementary Materials, Figure S4C,F). Within these regions, we performed digital segmentation of autolysosomes and quantified the area occupied by these organelles (Supplementary Materials, Figure S4D,G). This quantitative approach confirmed the trends observed in the low-magnification analysis (Supplementary Materials, Figure S4H). Thus, HC treatment primarily enhanced LC3 expression rather than dramatically increased the absolute number of autolysosomes.

To provide functional validation of the role of autophagy in mediating the effects of HC on renal cell proliferation, we performed experiments using autophagy inhibitors. 3-MA reduced the HC-induced increase in MTT-assessed cell viability, and CQ completely abolished it (Supplementary Materials, Figure S5), suggesting that the proliferative and/or cytoprotective effects of HC could be mediated by autophagy.

### 3.3. Systemic Effects of HC In Vivo

First, we evaluated the effects of HC on intact animals. Because HC is considered a caloric restriction mimetic [47], we monitored body weight throughout the entire experimental period. However, rats subjected to either 3-week or 7-week HC treatment showed no significant changes in body weight (Supplementary Materials, Figures S1 and S2). Since the effects of mimetics may be associated with reduced appetite [48], we assessed daily food intake and found no differences between the intact group and the animals administered HC. The absence of effects on body weight and food intake suggested that HC did not exert an appetite-suppressing effect at the selected therapeutic regimens.

### 3.4. Long-Term HC Administration Mitigated AKI Severity and Inflammatory Response

**Figure 4:**
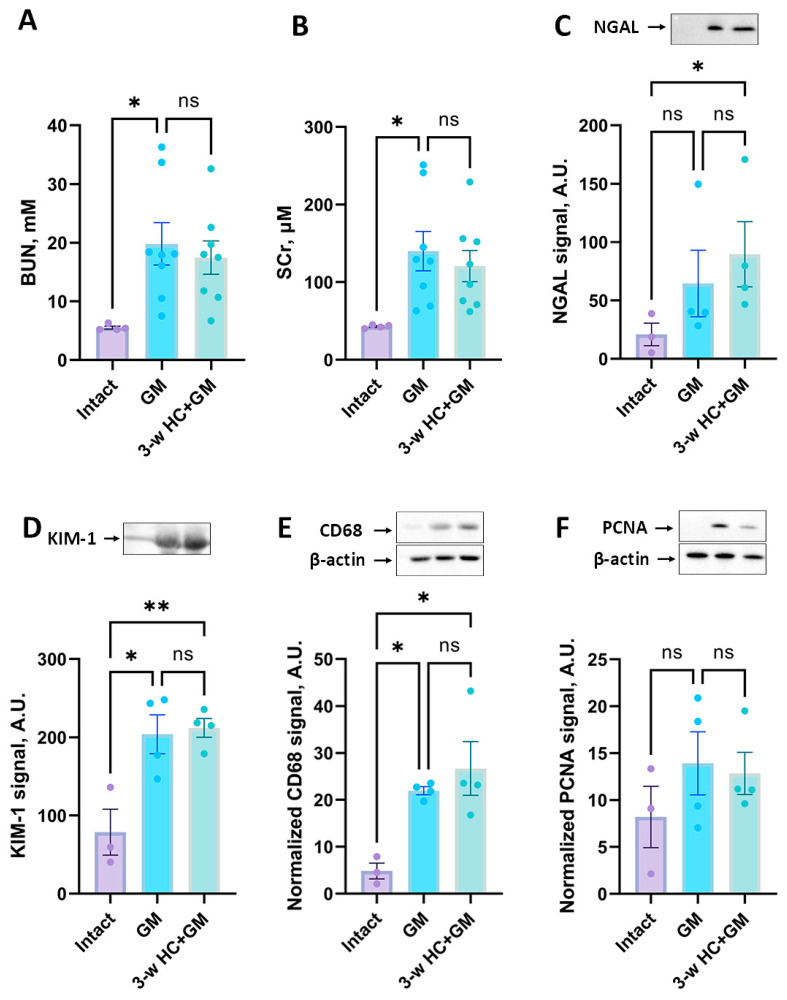
Influence of HC treatment for 3 weeks on the severity of AKI. The severity of AKI is evaluated by BUN (**A**) and SCr (**B**) concentrations. The comparison of NGAL (**C**) and KIM-1 (**D**) levels in the urine of rats. (**E**) CD68 levels in kidney tissue. (**F**) PCNA levels in kidney tissue. The original Western blot images are shown in Supplementary Materials, Figures S6–S8, correspondingly. * *p* < 0.05, ** *p* < 0.01, ns—not significant (one-way ANOVA with Tukey’s post hoc test).

It was previously shown that caloric restriction can exert protective effects in kidney pathologies, including ischemic ones [49,50]. In our study, we assessed the effect of 3- and 7-week HC treatment on the severity of gentamicin-induced AKI (Figure 1). We found that gentamicin administration for 6 days resulted in a significant increase in BUN and SCr levels, indicating AKI (Figure 4A,B).

The 3-week HC treatment prior to gentamicin administration showed no significant changes in renal failure compared to the non-treated gentamicin group (Figure 4A,B). In addition to BUN and SCr, we analyzed the levels of KIM-1 and NGAL as more sensitive markers of kidney damage. Gentamicin administration showed an increase in the urinary levels of these markers (Figure 4C,D), but we did not reveal any difference in urinary NGAL and KIM-1 levels between the “GM” and “3-w HC + GM” groups (Figure 4C,D). Although the mean values in the 3-week HC + GM group tended to be slightly higher than in the GM group for some parameters, these differences were not statistically significant and likely reflected biological variability. Thus, 3-week HC treatment failed to mitigate gentamicin-induced AKI.

**Figure 5:**
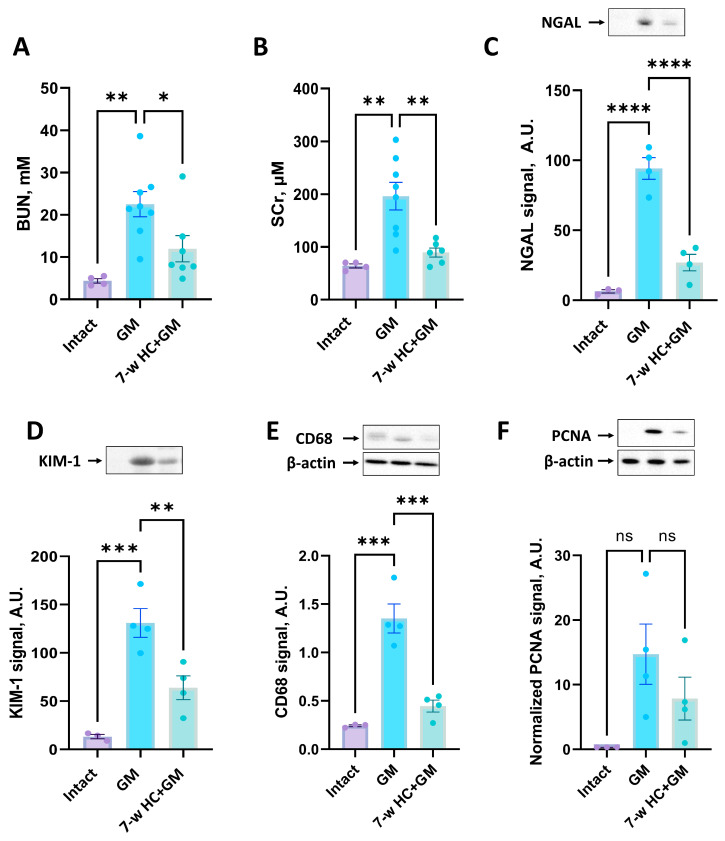
Influence of HC treatment for 7 weeks on the severity of AKI. The severity of AKI is evaluated by BUN (**A**) and SCr (**B**) concentrations. The comparison of NGAL (**C**) and KIM-1 (**D**) levels in the urine of rats. (**E**) CD68 levels in kidney tissue. (**F**) PCNA levels in kidney tissue. The original Western blot images are shown in Supplementary Materials, Figures S9–S11, correspondingly. * *p* < 0.05, ** *p* < 0.01, *** *p* < 0.001, **** *p* < 0.0001, ns—not significant (one-way ANOVA with Tukey’s post hoc test).

We further investigated the effect of prolonged 7-week HC treatment on the severity of gentamicin-induced AKI. We demonstrated a significant reduction in BUN and SCr levels (Figure 5A,B) and a statistically significant reduction in urinary NGAL and KIM-1 levels (Figure 5C,D) in the groups that received HC for 6 weeks prior to gentamicin administration. Thus, prophylactic HC administered for 7 weeks attenuated the severity of gentamicin-induced AKI.

We evaluated the level of CD68 protein as a marker of macrophages, which are key players in the immune response and inflammation. HC treatment for 3 weeks did not reduce the CD68 levels compared to the “GM” group (Figure 4E), indicating no effect on macrophage infiltration. In contrast, after 7 weeks of HC treatment, we observed a significant reduction in CD68 levels (Figure 5E), which suggests that 7 weeks of HC administration reduces macrophage infiltration and decreases inflammation.

The level of PCNA, a marker for cell proliferation, after 3 or 7 weeks of HC treatment showed no significant differences between treated and untreated groups, although it possessed a decreasing trend in AKI-induced PCNA in the “7-w HC + GM” group compared to “GM” group (Figure 4F and Figure 5F, correspondingly).

### 3.5. HC Increased Resistance to Apoptosis

**Figure 6:**
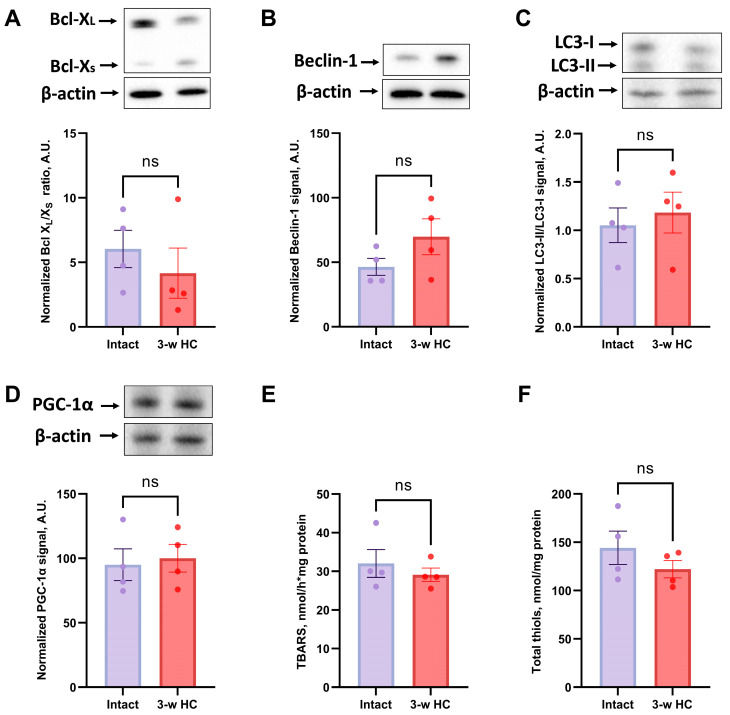
Evaluation of the effects of short-term HC administration for 3 weeks on the levels of anti-apoptotic and pro-apoptotic proteins, autophagy activation, mitochondrial biogenesis, and oxidative stress in kidney tissue. (**A**) Bcl-X_L_/X_S_ ratio; (**B**) Beclin-1 levels; (**C**) LC3-II/LC3-I ratio; (**D**) Levels of PGC-1α; (**E**) Measurement of TBARS; (**F**) Measurement of total thiols. The original Western blot images are shown in Supplementary Materials, Figures S12–S15, correspondingly. ns—not significant (Mann–Whitney test).

**Figure 7:**
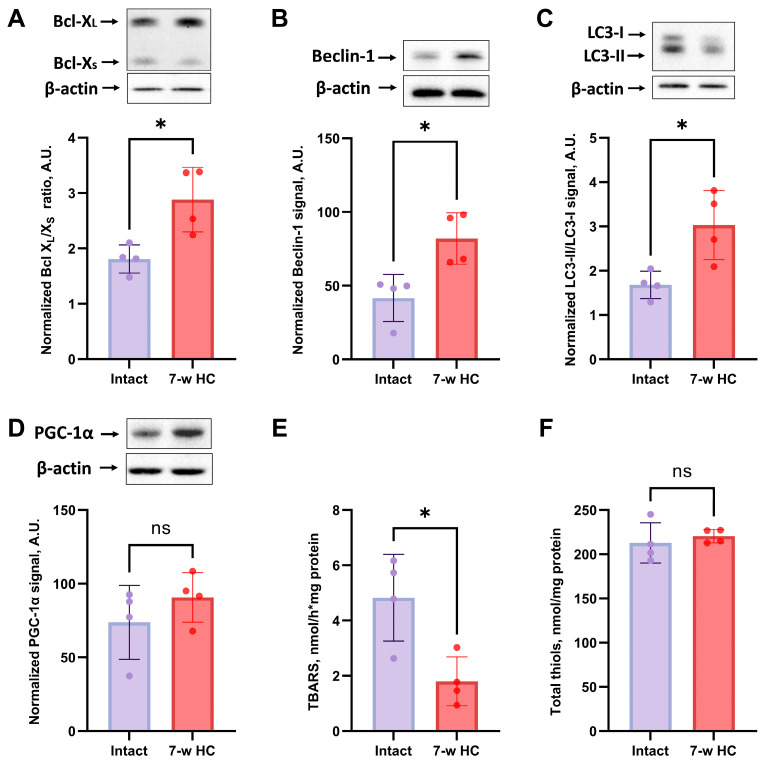
Evaluation of the effects of long-term HC administration for 7 weeks on the levels of anti-apoptotic and pro-apoptotic proteins, autophagy activation, mitochondrial biogenesis, and oxidative stress in kidney tissue. (**A**) Bcl-X_L_/X_S_ ratio; (**B**) Beclin-1 levels; (**C**) LC3-II/LC3-I ratio; (**D**) levels of PGC-1α; (**E**) measurement of TBARS; (**F**) measurement of total thiols. The original Western blot images are shown in the Supplementary Materials, Figures S16–S19, correspondingly. * *p* < 0.05, ns—not significant (Mann–Whitney test).

We examined the levels of anti-apoptotic protein Bcl-X_L_ and pro-apoptotic protein Bcl-X_S_ to explore the mechanisms of HC effects on cell survival and apoptosis regulation. Bcl-X_L_ and Bcl-X_S_ are involved in the mitochondrial pathway of apoptosis, and Bcl-X_L_/Bcl-X_S_ ratio reflects the anti-apoptotic capacity of the cell and the tissue [51]. In kidneys after 3 weeks of HC treatment, Bcl-X_L_/Bcl-X_S_ ratio showed no difference between HC-treated and intact rats (Figure 6A), but in the 7-week HC-treated rats the Bcl-X_L_/Bcl-X_S_ ratio showed a significant increase compared to the “Intact” group (Figure 7A). Thus, 7-week HC treatment significantly upregulated Bcl-X_L_ levels, suggesting an increased tolerance against gentamicin-induced apoptosis in kidney cells.

### 3.6. HC Activated Autophagy in Kidney

HC is thought to be an inducer and enhancer of autophagy [30]. Therefore, we assessed the LC3-II/LC3-I ratio and beclin-1 levels as markers and participants of the autophagy machinery. In vivo, HC demonstrated a significant increase in autophagy markers only after 7 weeks of administration, showing significant increases in both Beclin-1 levels and the LC3-II/LC3-I ratio (Figure 7B,C). However, 3-week HC treatment failed to induce any significant changes in Beclin-1 and the LC3-II/LC3-I ratio (Figure 6B,C), indicating no autophagy activation.

### 3.7. HC Affected Oxidative Stress Parameters Without Altering Mitochondrial Biogenesis Markers

We evaluated mitochondrial biogenesis through analysis of PGC-1α levels, which is also known to play an essential role in metabolic reprogramming in dietary interventions, coordinating the expression of genes involved in glucose and fatty acid metabolism [52]. HC treatment made no significant difference in PGC-1α levels whether 3 or 7 weeks of treatment were used (Figure 6 and Figure 7D).

To further elucidate the biochemical impacts of HC treatment, we assessed redox state and oxidative stress in kidney tissues by measuring Fe^2+^/ascorbate-induced TBARS and total thiol levels. An HC treatment of 7 weeks significantly reduced TBARS levels compared to the gentamicin-treated group (Figure 6E and Figure 7E); the treatment lasting 3 weeks did not. The result of the TBARS production confirmed the beneficial effects of HC treatment by reducing oxidative stress and enhancing antioxidant defenses in kidney tissues. Interestingly, the total thiols assay, which measures the overall thiol content in the cell, demonstrated no significant difference between the “Intact” and “3-w HC”, “7-w HC” groups (Figure 6F and Figure 7F).

## 4. Discussion

Despite significant advances in our understanding of the pathophysiology of AKI, effective pharmacological strategies for its prevention and treatment remain quite limited [53]. This challenge is particularly acute in the context of drug-induced nephrotoxicity, a common and serious iatrogenic complication associated with essential medications like aminoglycoside antibiotics [54]. Among these, gentamicin stands out as a widely used aminoglycoside with particularly high nephrotoxic potential, contributing substantially to the incidence of drug-induced AKI, which develops in up to 25% of patients [15]. The pathogenesis of gentamicin-induced nephrotoxicity is associated with the selective accumulation of the antibiotic in the proximal tubules, triggering the activation of proinflammatory cytokines and effector caspase-3, ultimately leading to the death of renal epithelial cells [41]. A key role in the realization of these processes is played by the dysregulation of the autophagic flux, as evidenced by the accumulation of the p62 protein [55]. This disruption leads to impaired mitochondrial function, including through the downregulation of *SIRT3* expression, and enhanced production of reactive oxygen species [56]. The resulting oxidative stress activates proinflammatory signaling cascades via NF-kB and increases the proportion of pro-apoptotic proteins (such as Bax), thereby triggering the mitochondrial pathway of apoptosis and exacerbating cell death [57,58].

Nevertheless, translating known molecular mechanisms into effective pharmacological strategies for the prevention or treatment gentamicin-induced nephrotoxicity continues to be a major challenge [59,60]. Therapeutic management remains largely supportive, with renal replacement therapy (RRT) serving as the primary intervention for severe cases, despite its invasive nature, hemodynamic complications, and inability to address the underlying cellular mechanisms of injury [61]. Current therapeutic strategies fail to directly target the core cellular mechanisms of injury, such as oxidative stress, inflammation, and dysregulated autophagy and apoptosis. This therapeutic gap underscores the urgent need for novel interventions that can protect the renal parenchyma. In this regard, strategies that mimic endogenous protective pathways, such as those activated by caloric restriction, represent a promising direction [21,47]. The exploration of caloric restriction mimetics offers a potential paradigm shift from supportive care to active cellular protection, aiming to enhance the kidney’s intrinsic resilience to toxic insults.

Indeed, caloric restriction has been shown to activate signaling pathways that counteract the mechanisms of gentamicin-induced injury. By reducing nutrient availability, caloric restriction inhibits mTOR and activates AMPK, a key energy sensor that promotes autophagy and maintains mitochondrial homeostasis [62]. Through mTOR inhibition, AMPK relieves the blockade of autophagy, facilitating the clearance of damaged organelles and proteins. In addition, caloric restriction upregulates the sirtuins SIRT1 and SIRT3, which are downregulated by gentamicin and play an important role in mitigating oxidative stress and mitochondrial dysfunction [63,64]. Additionally, it attenuates inflammation by suppressing proinflammatory cytokines such as IL-6 and TNF [65]. Thus, caloric restriction directly targets key pathogenic pathways in gentamicin-induced kidney damage. Given these protective effects, pharmacological agents that mimic caloric restriction without dietary intervention are of considerable interest. HC is one such mimetic, and the present study was designed to investigate its potential to reproduce these beneficial effects in the context of gentamicin-induced nephrotoxicity.

While the molecular mechanisms underlying the renoprotective effects of HC remain poorly understood, its biological activity in other experimental contexts suggests that HC exerts diverse effects beyond ATP-citrate lyase inhibition. HC has been shown to improve hypertension, dyslipidemia and diabetes [66], inhibit kidney stone formation [67] and reduce tumor growth via AMP-activated protein kinase (AMPK)/mechanistic target of rapamycin (mTOR) activation [68]. Previous studies in rodents have primarily investigated long-term administration of HC (≥10 weeks), demonstrating benefits such as lower body weight, improved metabolic parameters and a reduction in oxidative stress in kidney and liver tissue [69,70,71,72,73,74,75]. Similar effects were also observed in other organs, including reduced lipid peroxidation and inflammation in the liver after 7 weeks of HC administration [76]. However, prolonged HC use (4 months) has also been associated with paradoxical effects, such as collagen accumulation in the liver and an increase in oxidative stress markers [69].

HC has shown inconsistent effects on weight loss, including in humans. Some studies reported modest benefits from serotonin-mediated appetite suppression [77,78], while others found no significant effect [76,79]. These inconsistencies may be due to differences in dosing, treatment duration or metabolic context. In our study, neither the 3-week nor the 7-week HC treatment had a significant effect on body weight or food intake in rats (Supplementary Materials, Figures S1 and S2), which is consistent with reports questioning the efficacy of HC for weight loss [69,80]. This deviation from the positive results in chronic models [76,79] may reflect dose- or time-dependent effects, suggesting that short-term administration of HC does not reproduce the metabolic adaptations observed with longer protocols.

While HC has been extensively studied for its metabolic effects, its influence on AKI remains unclear, as do the optimal duration and design of HC treatment to prevent or treat AKI, with limited clinical data [81]. Given the limited literature on the protective effects of HC in acute injury, our study aims to address this gap by systematically evaluating its effects on oxidative stress, inflammation, autophagy and apoptosis in AKI, while clarifying discrepancies in reported metabolic effects. In this study, our focus was not on metabolic modulation but on the nephroprotective potential and the critical role of treatment duration in HC-mediated protection against AKI.

To investigate the potential protective effects of HC, we first performed in vitro experiments. Our results showed that HC at concentrations of 0.125 to 2 mM had a strong promoting effect on kidney cell proliferation and viability on both NRK-52E and MDCK cell lines (Figure 2; Supplementary Materials, Figure S3). To uncover the possible molecular mechanisms of HC action that could be responsible for the positive effects, we then analyzed the activation of the autophagic system as an important repair mechanism in both cultured kidney cells and kidney tissue after HC treatment. For this purpose, Cyto-ID staining was performed after incubation with HC to reveal the activation of autophagosome formation. Cyto-ID quantification showed greater activation of autophagy after treatment with 1 mM HC compared to untreated cells (Figure 3B). Moreover, we used MDCK cells expressing mCherry-GFP-LC3 to prove the results obtained (Figure 3C). We observed that the majority of LC3-positive organelles were mCherry-positive but GFP-negative, suggesting that these organelles are autolysosomes. This observation aligns with the short lifespan of a mature, closed, yet still neutral autophagosome before significant acidification or fusion with the lysosome, whereas autolysosomes persist longer during cargo degradation [82,83]. Experiments with cells stably expressing LC3-GFP-mCherry showed an increase in LC3 expression after treatment with 1 mM HC, rather than increasing the absolute number of autophagosomes or autolysosomes (Figure 3E–G; Supplementary Materials, Figure S4). Furthermore, we showed that the inhibitor of late stages of autophagy CQ revoked HC-induced activation of cell proliferation (Supplementary Materials, Figure S5). These data provide a direct mechanistic link between autophagy and HC-induced effects in the kidney. We also assessed autophagy activation in rats and showed that 7-week HC administration increased LC3-II/LC3-I ratio and beclin-1 levels in kidney tissue (Figure 7B,C). The fact that autophagy was upregulated after 7 weeks, but not after 3 weeks of treatment supports the notion that HC may gradually prime autophagy pathways to promote cellular clearance of damaged components, thereby facilitating renal recovery rather than providing immediate cytoprotection.

Although previous studies suggested that HC affects autophagic pathways that mimic the effects of caloric restriction [84,85], evidence supporting HC-induced autophagy remains limited and context-specific. Previous studies have demonstrated HC-mediated autophagy activation in S. cerevisiae [86] and increased antitumor response by inducing autophagy and reprogramming the tumor immune microenvironment, shifting the balance from immunosuppressive M2-like phenotypes toward proinflammatory M1-like activation, thereby enhancing local immunosurveillance [87]. Several other publications demonstrated the pro-autophagic potential of HC but did not validate it in the context of AKI [31,47,88], and none have considered treatment duration—a factor our data identify as critical.

Building on our promising results in vitro, we conducted animal studies to further investigate the nephroprotective potential of HC in a gentamicin-induced AKI model, testing two distinct treatment durations to evaluate their efficacy in ameliorating gentamicin-induced AKI. To clarify this, we investigated the effects of HC on AKI markers, oxidative stress and cellular repair pathways. Gentamicin-induced AKI was significantly attenuated by 7 weeks of HC treatment, as evidenced by a reduction in BUN and SCr (Figure 5A,B). These functional improvements correlated with a reduction in oxidative stress, as shown by the decrease in the TBARS formation induced by Fe^2+^/ascorbate (Figure 7E), emphasizing that the antioxidant capacity of HC requires sustained administration. In contrast, 3-week administration of HC failed to reduce oxidative damage, reinforcing the conclusion that prolonged HC exposure is necessary for effective renal protection [69,80].

A critical finding of our study is that treatment duration is a key determinant of the therapeutic efficacy and anti-inflammatory action of HC. Inflammation, a major cause of AKI [89], was attenuated by 7-week HC, as shown by reduced infiltration of CD68+ macrophages (Figure 5E). This is consistent with the documented anti-inflammatory properties of HC [23,76,90] and may be associated with reduced damage to kidney cells during injury due to increased tolerance. In addition, the inflammation-modulating effect of HC observed in our study may be related to the alteration in autophagy and oxidative stress. First, the HC-induced autophagy activation, particularly evident after 7 weeks of treatment (Figure 7B,C), may contribute to the regulation of renal inflammatory responses. Autophagy is tightly controlled by the AMPK/mTOR signaling axis, a central regulator of both cellular metabolism and immune cell function. Activation of AMPK [91] and inhibition of mTOR [92] have been shown to promote a shift in macrophages from a proinflammatory (M1) phenotype to an anti-inflammatory (M2) phenotype, thereby supporting tissue repair rather than sustaining inflammation [93,94]. Although macrophage polarization was not directly assessed in the present study, enhanced autophagic activity after 7-week HC treatment (Figure 7B,C) may represent one of the mechanisms contributing to the attenuation of renal inflammation. Second, oxidative stress, particularly mitochondrial reactive oxygen species, is a well-established activator of NOD-like receptor protein 3 (NLRP3) inflammasome, which promotes inflammation [95]. In this context, the reduction in lipid peroxidation products observed after HC treatment indicates an improved redox balance and reduced oxidative damage. Such redox modulation may limit proinflammatory signaling cascades associated with oxidative stress and inflammasome activation [95], thereby contributing to the anti-inflammatory effects of prolonged HC administration.

HC also showed anti-apoptotic effects [96], as confirmed by our data, with 7 weeks of treatment increasing the Bcl-X_L_/Bcl-X_S_ ratio (Figure 7A), which likely contributed to tubule cell survival. Surprisingly, PGC-1α, a regulator of mitochondrial biogenesis, remained unchanged (Figure 6D and Figure 7D), suggesting that the benefits of HC are mediated by direct antioxidant and anti-apoptotic mechanisms rather than changes in mitochondrial biogenesis. It should be noted that the preventive regimens differed in both duration and dosage. Although the 7-week regimen has proven effective, the current regimen does not allow complete separation of treatment duration from dosage. Future studies testing the influence of individual factors are needed to isolate these variables.

## 5. Conclusions

In this study, we show that the caloric restriction mimetic hydroxycitrate exerts nephroprotective effects in a gentamicin-induced acute kidney injury model, but this protection depends strongly on the duration of treatment. Short-term (3-week) administration had no detectable protective effect, while prolonged 7-week treatment significantly attenuated renal dysfunction and reduced markers of kidney injury. The protective effects of HC were associated with activation of autophagy, reduced oxidative stress, decreased macrophage infiltration, and increased resistance of renal cells to apoptosis, indicating a coordinated cytoprotective response in kidney tissue. These findings suggest that hydroxycitrate may be a promising strategy for preventing drug-induced nephrotoxicity. Future studies should investigate longer treatment durations and higher doses to determine whether the metabolic and renal benefits of HC coincide in models of chronic kidney disease.

## Data Availability

The data that support the findings of this study are available from the corresponding author upon reasonable request.

## Supplementary Materials

The following supporting information can be downloaded at: https://www.mdpi.com/article/10.3390/biom16040538/s1, Figure S1. The effects of the short-term regimen of HC administration on body weight and food intake. Figure S2. The effects of the long-term regimen of HC administration on body weight and food intake. Figure S3. Effects of HC on the viability of MDCK cells in vitro. Figure S4. The analysis of autolysosome content in vitro in kidney cells in response to incubation with HC. Figure S5. Effect of autophagy inhibitors on HC-induced increase in cell proliferation assessed by MTT assay. Figure S6. Raw uncropped western blot images of NGAL (raw image to Figure 4C) and KIM-1 levels (raw image to Figure 4D) in the urine; short-term regimen (3-week HC administration). Figure S7. Raw uncropped western blot images of CD68 levels in kidney homogenates (raw image to Figure 4E); short-term regimen (3-week HC administration). Figure S8. Raw uncropped western blot images of PCNA levels in kidney homogenates (raw image to Figure 4F); short-term regimen (3-week HC administration). Figure S9. Raw uncropped western blot images of NGAL (raw image to Figure 5C) and KIM-1 levels (raw image to Figure 5D) in the urine; long-term regimen (7-week HC administration). Figure S10. Raw uncropped western blot images of CD68 levels in kidney homogenates (raw image to Figure 5E); long-term regimen (7-week HC administration). Figure S11. Raw uncropped western blot images of PCNA levels in kidney homogenates (raw image to Figure 5F); long-term regimen (7-week HC administration). Figure S12. Raw uncropped western blot images of Bcl-X_L_ and Bcl-X_S_ levels in kidney homogenates (raw image to Figure 6A); short-term regimen (3-week HC administration). Figure S13. Raw uncropped western blot images of beclin-1 levels in kidney homogenates (raw image to Figure 6B); short-term regimen (3-week HC administration). Figure S14. Raw uncropped western blot images of LC3-II and LC3-I levels in kidney homogenates (raw image to Figure 6C); short-term regimen (3-week HC administration). Figure S15. Raw uncropped western blot images of PGC-1α levels in kidney homogenates (raw image to Figure 6D); short-term regimen (3-week HC administration). Figure S16. Raw uncropped western blot images of Bcl-X_L_ and Bcl-X_S_ levels in kidney homogenates (raw image to Figure 7A); long-term regimen (7-week HC administration). Figure S17. Raw uncropped western blot images of beclin-1 levels in kidney homogenates (raw image to Figure 7B); long-term regimen (7-week HC administration). Figure S18. Raw uncropped western blot images of LC3-II and LC3-I levels in kidney homogenates (raw image to Figure 7C); long-term regimen (7-week HC administration). Figure S19. Raw uncropped western blot images of PGC-1α levels in kidney homogenates (raw image to Figure 7D); long-term regimen (7-week HC administration).

## Abbreviations

The following abbreviations are used in this manuscript:

AKI	acute kidney injury
AMPK	AMP-activated protein kinase
Bcl-X_L_	B-cell lymphoma-extra large
Bcl-X_s_	B-cell lymphoma-extra small
BUN	blood urea nitrogen
CD68	cluster of differentiation 68
HC	hydroxycitrate
SCr	serum creatinine
DMSO	dimethyl sulfoxide
DPBS	Dulbecco’s phosphate-buffered saline
FBS	fetal bovine serum
GFP	green fluorescent protein
KIM-1	kidney injury molecule-1
LC3	light chain 3
mTOR	mechanistic target of rapamycin
NGAL	neutrophil gelatinase-associated lipocalin
PBS	phosphate-buffered saline
PCNA	proliferating cell nuclear antigen
PGC-1α	proliferator-activated receptor gamma coactivator 1-alpha
PMSF	phenylmethylsulfonyl fluoride
TBARS	thiobarbituric acid-reactive substances
RRT	Renal Replacement Therapy
