# Supplementary material for: Caloric Restriction Mimetic Hydroxycitrate Mitigates Acute Nephrotoxicity via Autophagy Activation and Oxidative Stress Reduction
Source: Biomolecules. 2026 Apr 4;16(4):538. doi: 10.3390/biom16040538 (PMC13113946; doi:10.3390/biom16040538)
Supplement: Supplementary file 1 [file biomolecules-16-00538-s001.zip › biomolecules-4171228-supplementary.pdf]

Caloric restriction mimetic hydroxycitrate mitigates acute nephrotoxicity via autophagy activation and oxidative stress reduction

Xinyu Liao, Nadezda V. Andrianova, Ljubava D. Zorova, Anna A. Brezgunova, Kseniia S. Cherkesova, Marina I. Buyan, Dmitry S. Semenovich, Alexandra A. Dalina, Irina B. Pevzner, Juan Jin, Yunguang Wang, Egor Y. Plotnikov

Supplementary figures

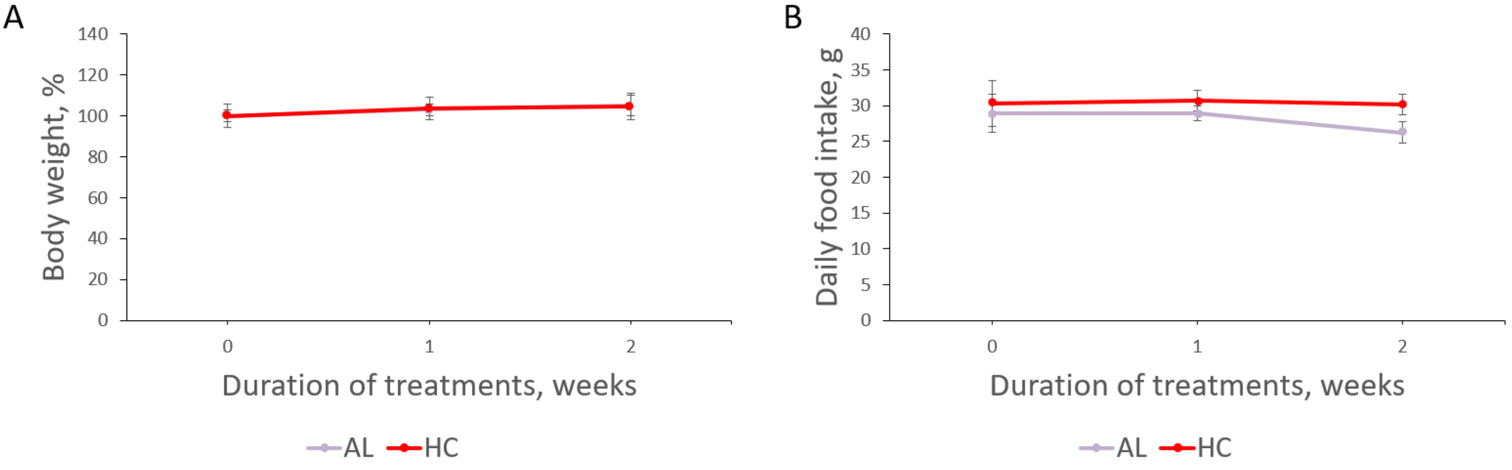

Supplementary Fig. S1. The effects of the short-term regimen of HC administration on body weight and food intake. (A) Percentage change in body weight of rats after first 2 weeks of HC treatment; (B) Daily food consumption in rats after 2 weeks of HC treatment.

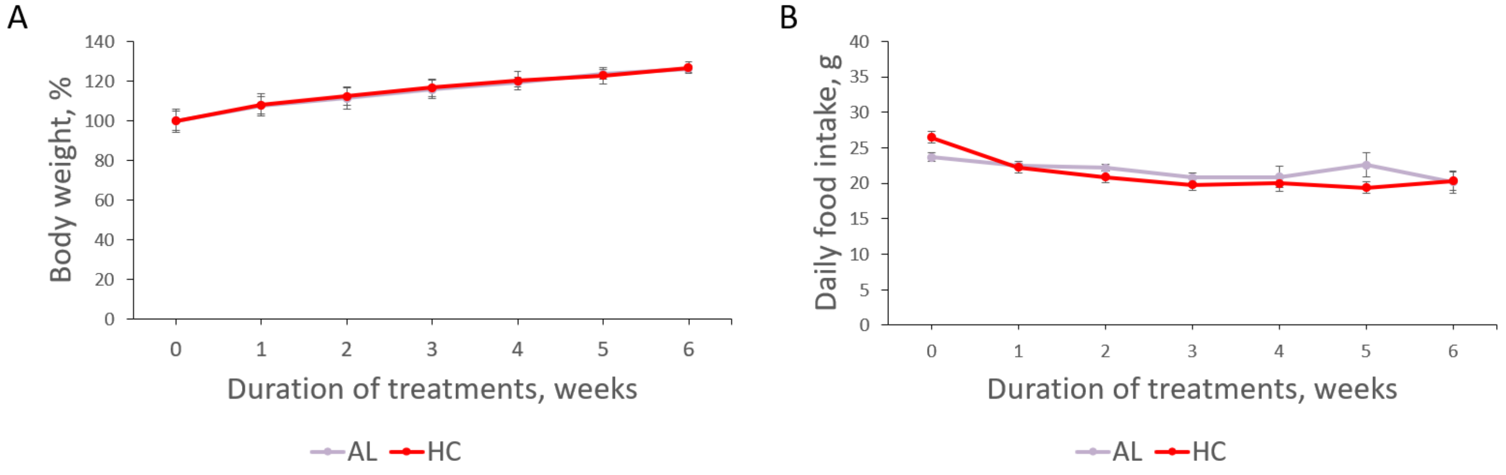

Supplementary Fig. S2. The effects of the long-term regimen of HC administration on body weight and food intake. (A) Percentage change in body weight of rats after first 6 weeks of HC treatment; (B) Daily food consumption in rats after 6 weeks of HC treatment.

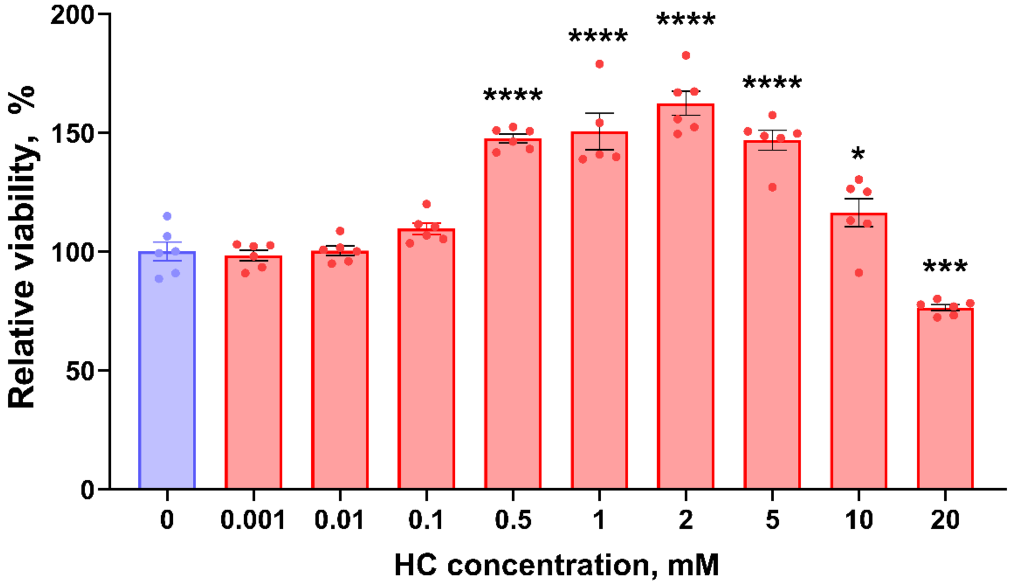

Supplementary Fig. S3. Effects of HC on the viability of MDCK cells *in vitro*. Number of viable MDCK cells were assessed by MTT assay after incubation with different concentrations of HC. *$p < 0.05$, ***$p < 0.001$, ****$p < 0.0001$ compared to the control group (one-way ANOVA with Tukey's post-hoc test).

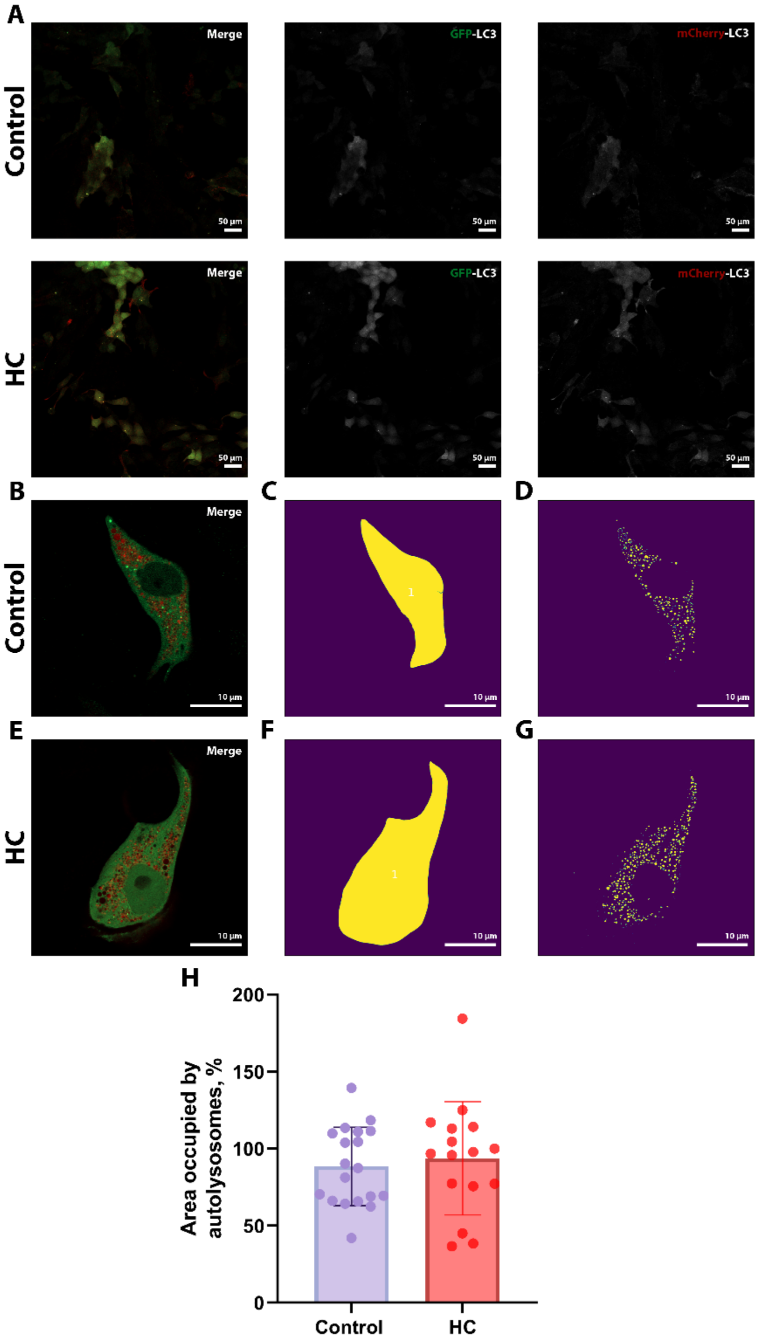

Supplementary Fig. S4. The analysis of autolysosome content in vitro in kidney cells in response to incubation with HC. (A) Representative confocal images showing mCherry and GFP fluorescence in control and HC-treated MDCK cells at low magnification. Scale bar, 50 µm. (B) Representative confocal image of a control MDCK cell transfected with the mCherry-GFP-LC3 reporter at high magnification. Scale bar, 10 µm. (C) Cell area mask for a control transfected MDCK cell. (D) Intercellular compartment mask detecting autolysosomes in a control transfected MDCK cell. (E) Representative confocal image of an MDCK cell transfected with the mCherry-GFP-LC3 reporter after incubation with HC, imaged at high magnification. Scale bar, 10 µm. (F) Cell area mask for a transfected MDCK cell after incubation with HC. (G) Intercellular compartment mask detecting autolysosomes in a transfected MDCK cell after incubation with HC. (H) Quantification of the relative area occupied by autolysosomes, expressed as a percentage of the total cell area.

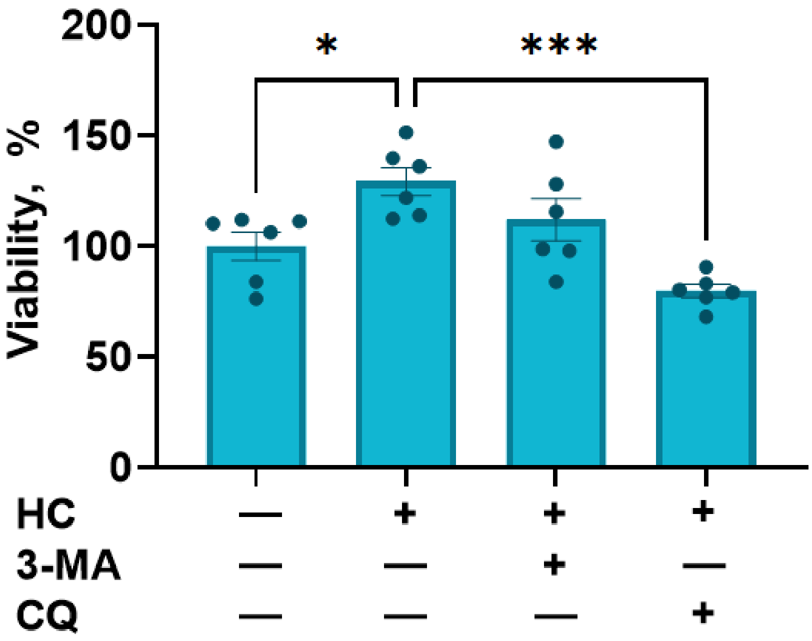

Supplementary Fig. S5. Effect of autophagy inhibitors on HC-induced increase in cell proliferation assessed by MTT assay. NRK-52E cells were treated with HC (0.5 mM), 3-methyladenine (3-MA, 2.5 mM) or chloroquine (CQ, 40 μM) as indicated. *$p < 0.05$, ***$p < 0.001$ (one-way ANOVA followed by Tukey's post hoc test).

NGAL (Figure 4 C)

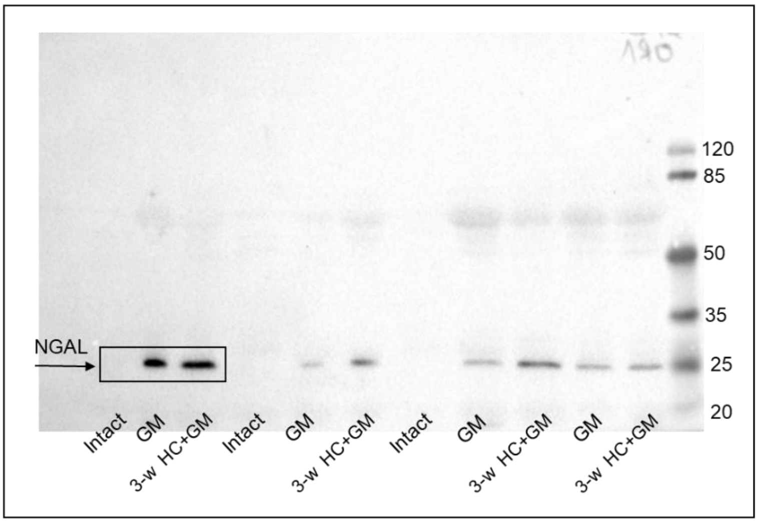

KIM-1 (Figure 4 D)

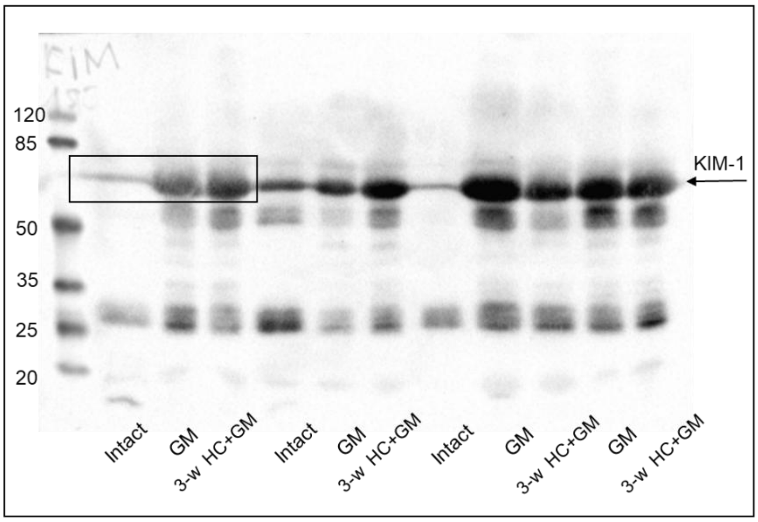

Supplementary Fig. S6. Raw uncropped western blot images of NGAL (raw image to Fig. 4C) and KIM-1 levels (raw image to Fig. 4D) in the urine; short-term regimen (3-week HC administration).

CD68 (Figure 4 E)

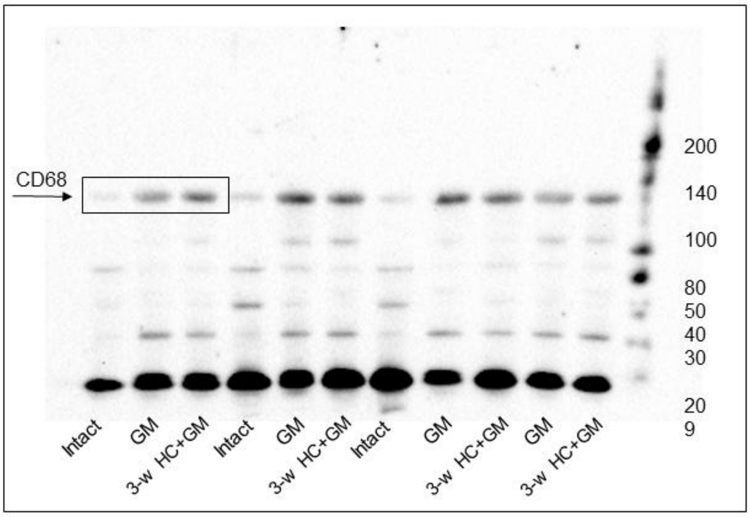

β-actin to CD68 (Figure 4 E)

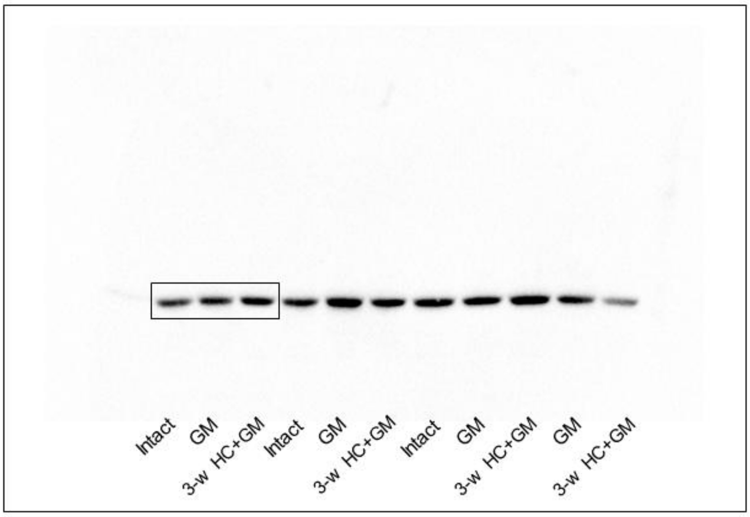

Supplementary Fig. S7. Raw uncropped western blot images of CD68 levels in kidney homogenates (raw image to Fig. 4E); short-term regimen (3-week HC administration).

PCNA (Figure 4 F)

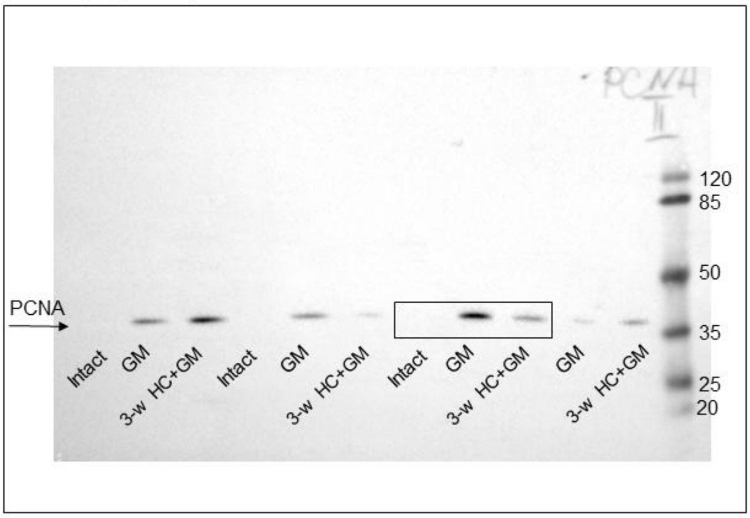

β-actin to PCNA (Figure 4 F)

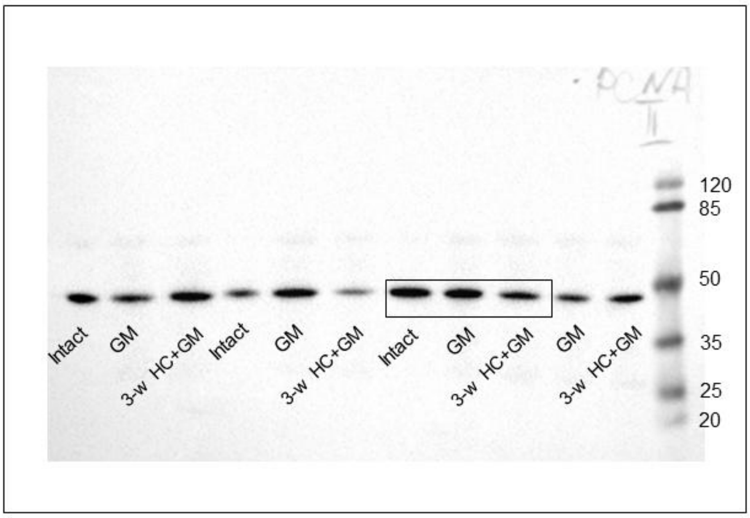

Supplementary Fig. S8. Raw uncropped western blot images of PCNA levels in kidney homogenates (raw image to Fig. 4F); short-term regimen (3-week HC administration).

NGAL (Figure 5 C)

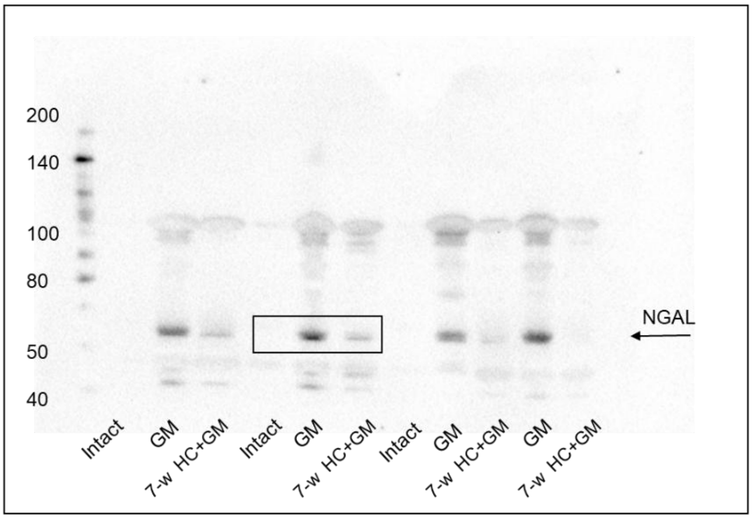

KIM-1 (Figure 5 D)

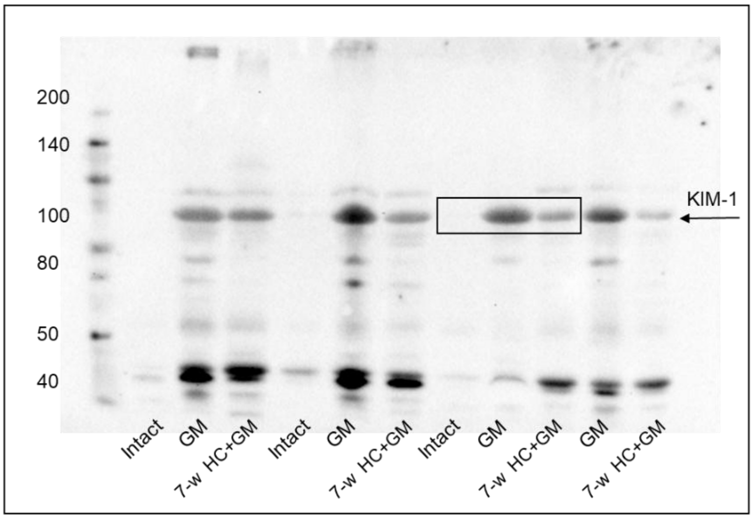

Supplementary Fig. S9. Raw uncropped western blot images of NGAL (raw image to Fig. 5C) and KIM-1 levels (raw image to Fig. 5D) in the urine; long-term regimen (7-week HC administration).

CD68 (Figure 5 E)

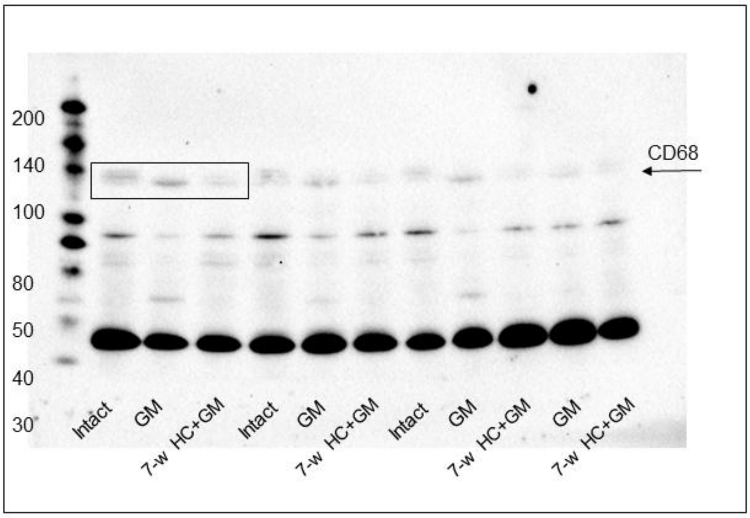

β-actin to CD68 (Figure 5 E)

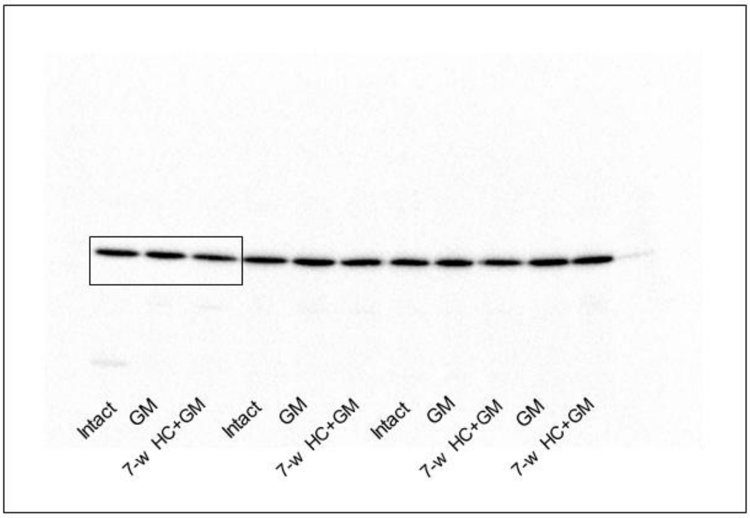

Supplementary Fig. S10. Raw uncropped western blot images of CD68 levels in kidney homogenates (raw image to Fig. 5E); long-term regimen (7-week HC administration).

PCNA (Figure 5 F)

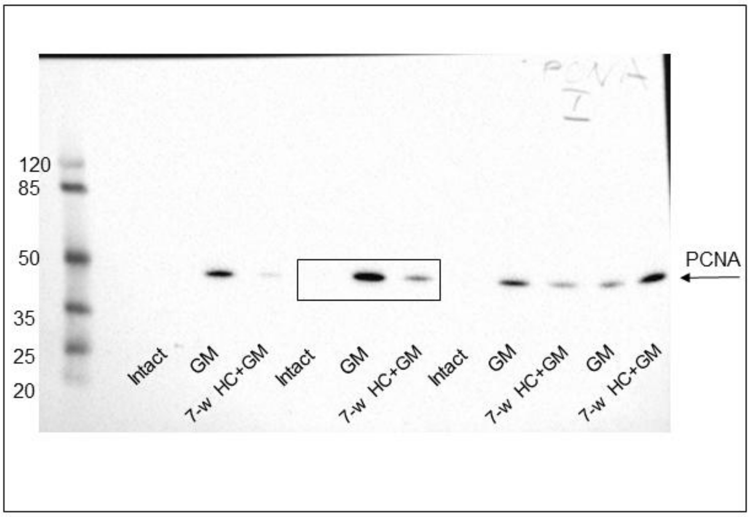

β-actin to PCNA (Figure 5 F)

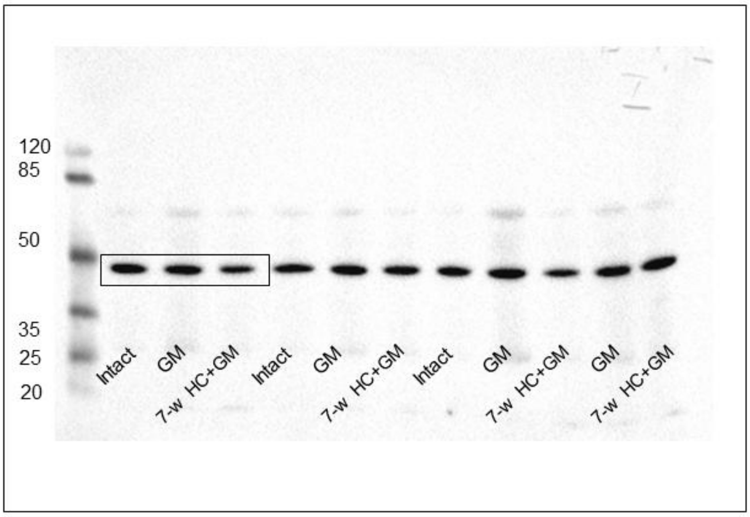

Supplementary Fig. S11. Raw uncropped western blot images of PCNA levels in kidney homogenates (raw image to Fig. 5F); long-term regimen (7-week HC administration).

BCL-X (Figure 6 A)

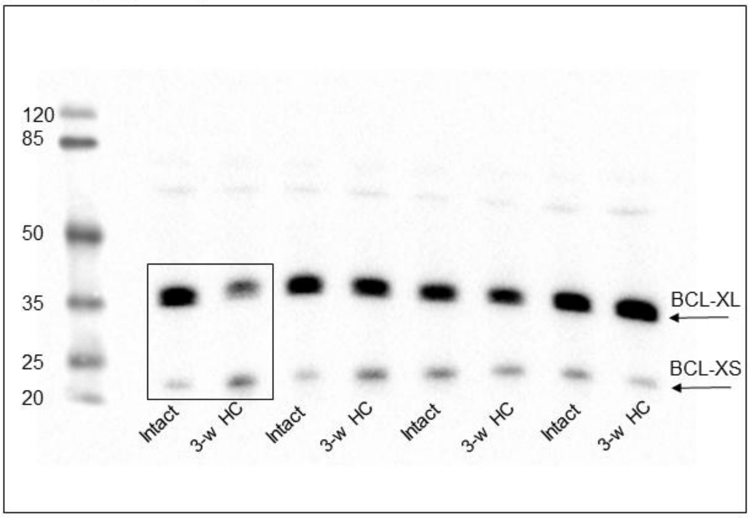

β-actin to BCL-X (Figure 6 A)

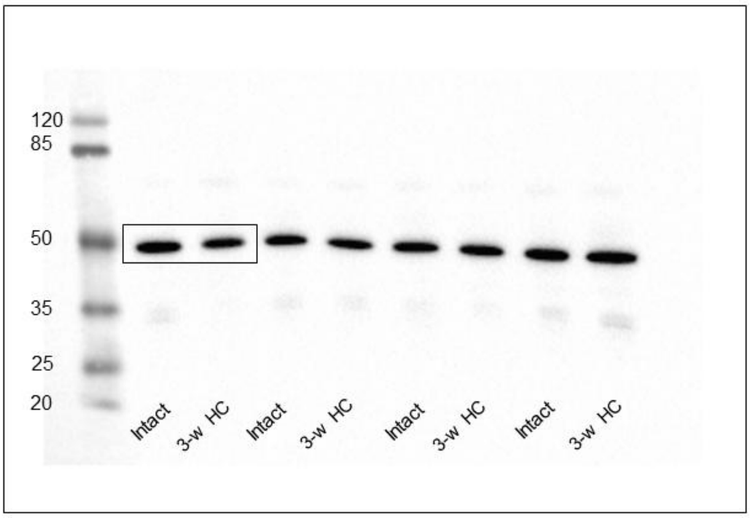

Supplementary Fig. S12. Raw uncropped western blot images of Bcl-X_L and Bcl-X_S levels in kidney homogenates (raw image to Fig. 6A); short-term regimen (3-week HC administration).

Beclin-1 (Figure 6 B)

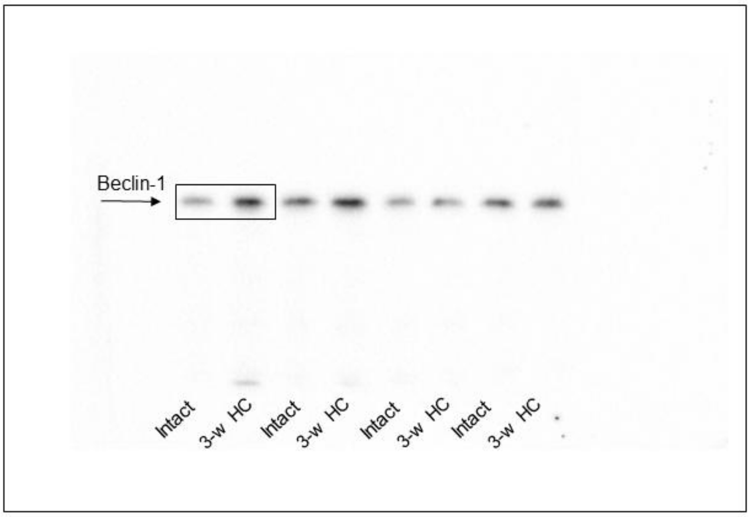

β-actin to Beclin-1 (Figure 6 B)

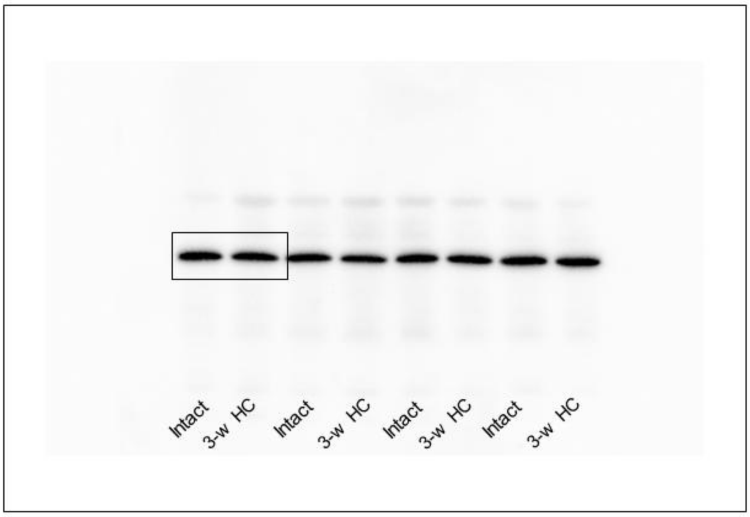

Supplementary Fig. S13. Raw uncropped western blot images of beclin-1 levels in kidney homogenates (raw image to Fig. 6B); short-term regimen (3-week HC administration).

LC3 (Figure 6 C)

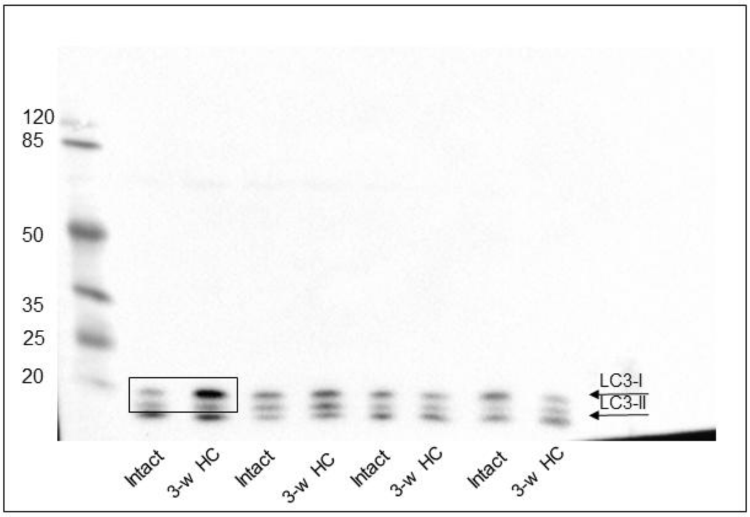

β-actin to LC3 (Figure 6 C)

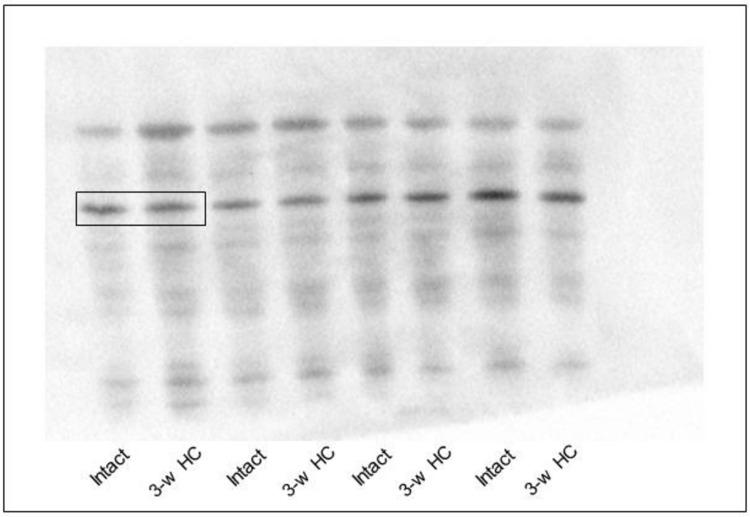

Supplementary Fig. S14. Raw uncropped western blot images of LC3-II and LC3-I levels in kidney homogenates (raw image to Fig. 6C); short-term regimen (3-week HC administration).

PGC-1α (Figure 6 D)

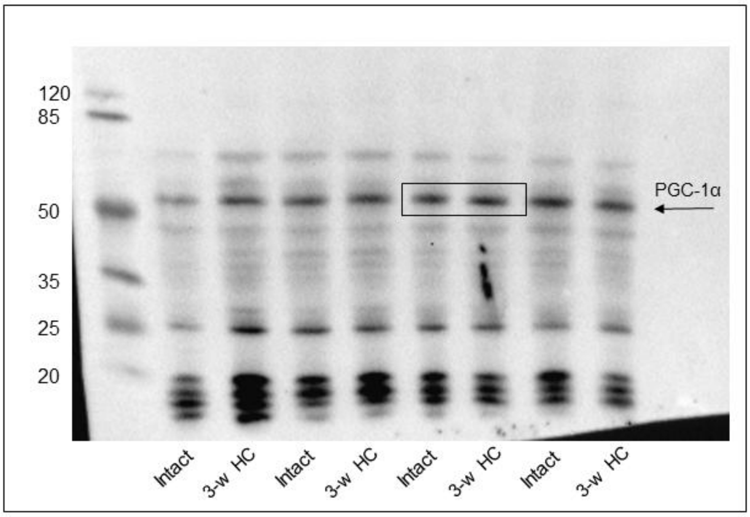

β-actin to PGC-1α (Figure 6 D)

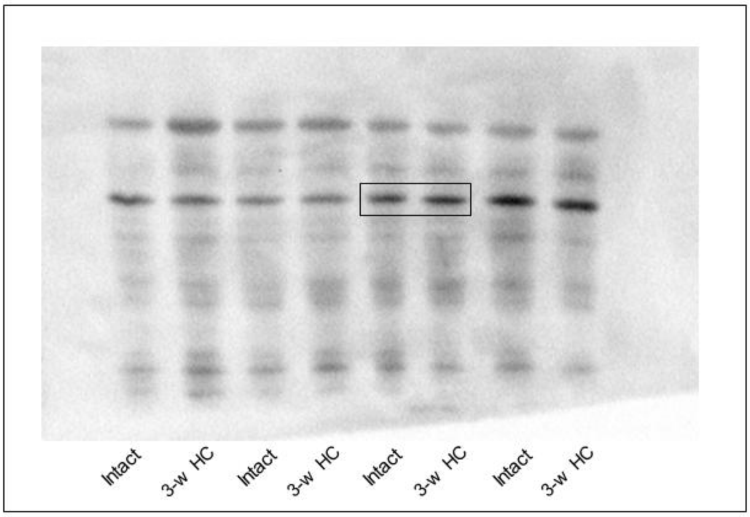

Supplementary Fig. S15. Raw uncropped western blot images of PGC-1α levels in kidney homogenates (raw image to Fig. 6D); short-term regimen (3-week HC administration).

BCL-X (Figure 7 A)

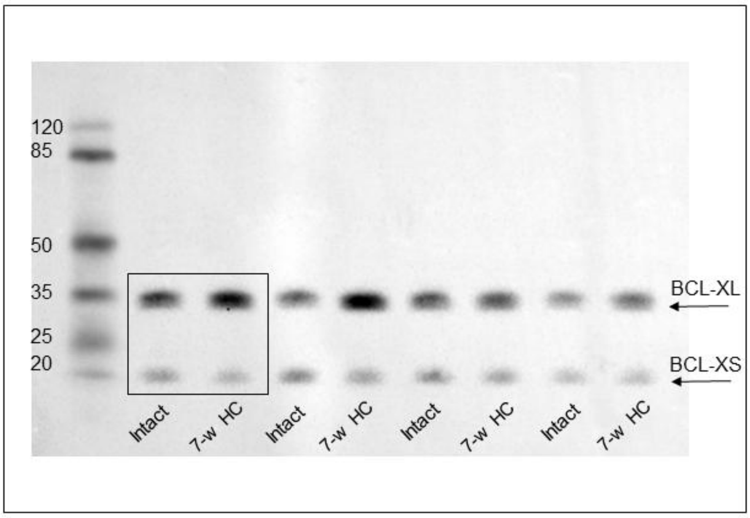

β-actin to BCL-X (Figure 7 A)

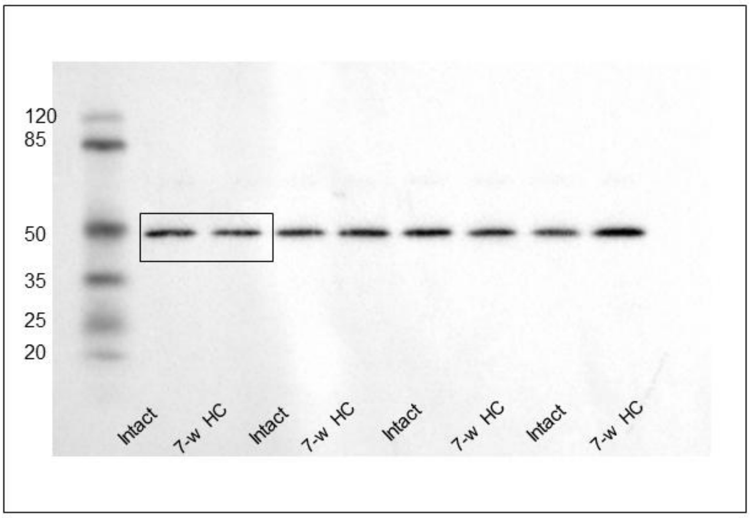

Supplementary Fig. S16. Raw uncropped western blot images of Bcl-X$_L$ and Bcl-X$_S$ levels in kidney homogenates (raw image to Fig. 7A); long-term regimen (7-week HC administration).

Beclin-1 (Figure 7 B)

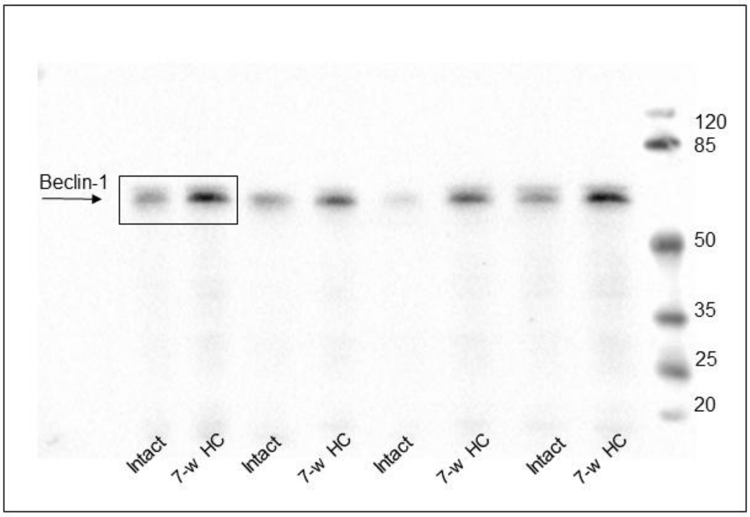

β-actin to Beclin-1 (Figure 7 B)

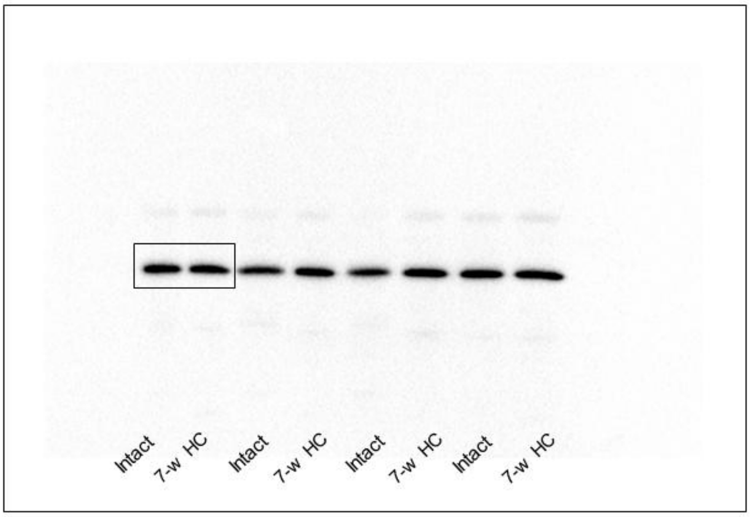

Supplementary Fig. S17. Raw uncropped western blot images of beclin-1 levels in kidney homogenates (raw image to Fig. 7B); long-term regimen (7-week HC administration).

LC3 (Figure 7 C)

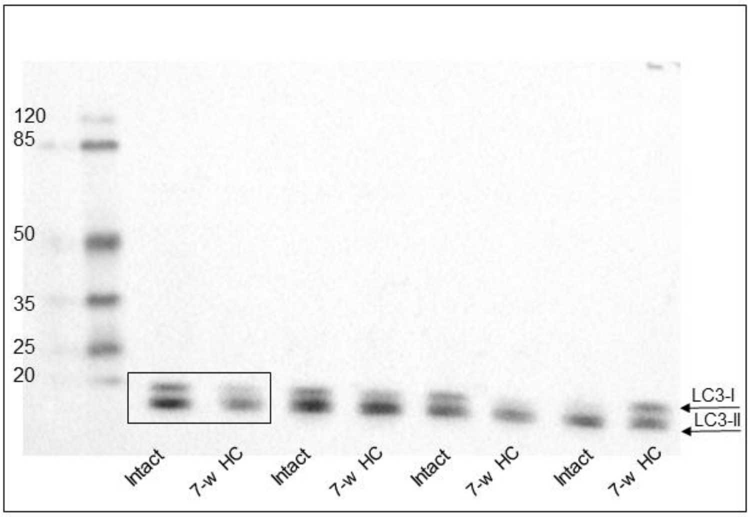

β-actin to LC3 (Figure 7 C)

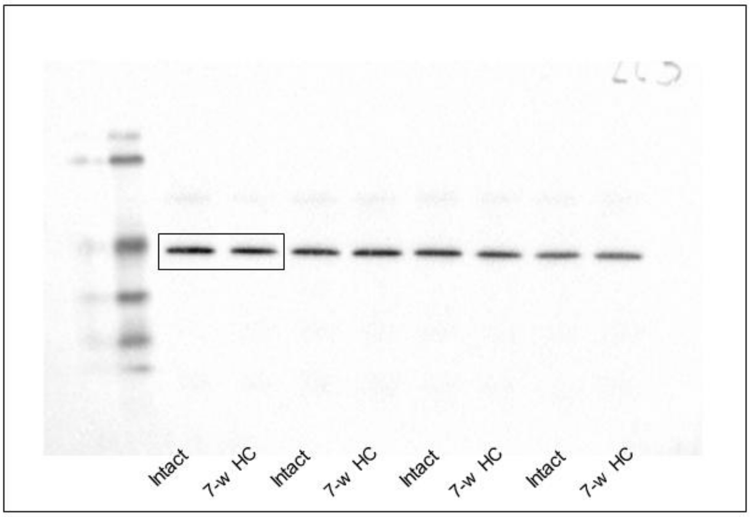

Supplementary Fig. S18. Raw uncropped western blot images of LC3-II and LC3-I levels in kidney homogenates (raw image to Fig. 7C); long-term regimen (7-week HC administration).

PGC-1α (Figure 7 D)

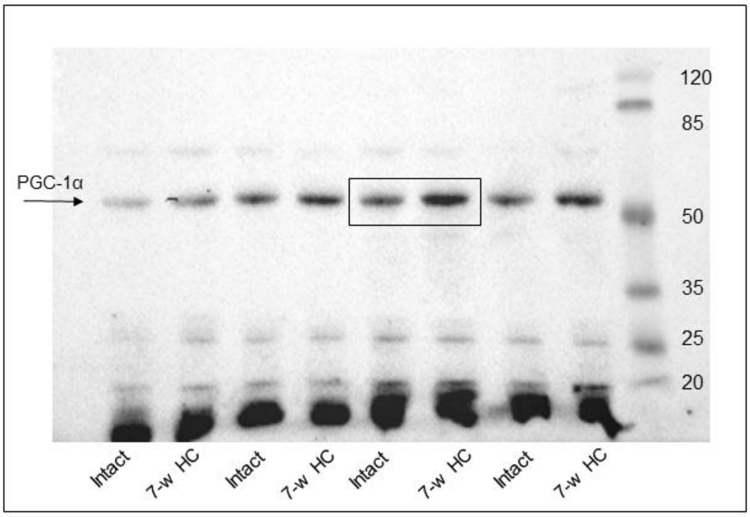

β-actin to PGC-1α (Figure 7 D)

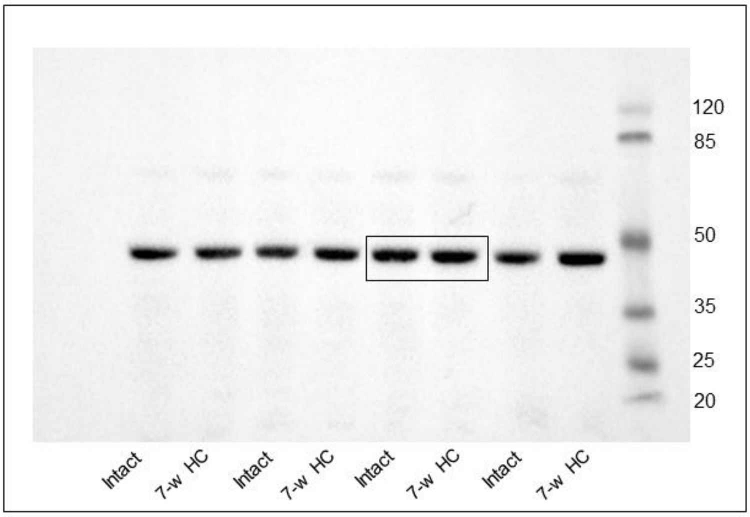

Supplementary Fig. S19. Raw uncropped western blot images of PGC-1α levels in kidney homogenates (raw image to Fig. 7D); long-term regimen (7-week HC administration).
